# Myocarditis Following COVID-19 Vaccination: Cardiac Imaging Findings in 118 Studies

**DOI:** 10.3390/tomography8040164

**Published:** 2022-07-30

**Authors:** Pedram Keshavarz, Fereshteh Yazdanpanah, Maryam Emad, Azadeh Hajati, Seyed Faraz Nejati, Faranak Ebrahimian Sadabad, Tamta Azrumelashvili, Malkhaz Mizandari, Steven S. Raman

**Affiliations:** 1 Department of Radiological Sciences, David Geffen School of Medicine, University of California, Los Angeles (UCLA), Los Angeles, CA 90095, USA; pkeshavarz@mednet.ucla.edu (P.K.); sraman@mednet.ucla.edu (S.S.R.); 2 School of Science and Technology, The University of Georgia, Tbilisi 0171, Georgia; 3 Network of Immunity in Infection, Malignancy and Autoimmunity (NIIMA), Universal Scientific Education and Research Network (USERN), Tabriz 5166, Iran; fereshteh.yazdanpanah89@gmail.com; 4 Taba Medical Imaging Center, Shiraz 71347-53151, Iran; mym.emad@gmail.com (M.E.); azadeh.hajati@gmail.com (A.H.); faraz.nejati@yahoo.com (S.F.N.); faranak.ebrahimian@yahoo.com (F.E.S.); 5 Department of Diagnostic & Interventional Radiology, New Hospitals Ltd., Tbilisi 0114, Georgia; tamta_azrumelashvili@yahoo.com

**Keywords:** coronavirus, myocarditis, Pfizer-BioNTech, Moderna, Janssen/Johnson & Johnson

## Abstract

We reviewed the reported imaging findings of myocarditis in the literature following COVID-19 vaccination on cardiac imaging by a literature search in online databases, including Scopus, Medline (PubMed), Web of Science, Embase (Elsevier), and Google Scholar. In total, 532 cases of myocarditis after COVID-19 vaccination were reported (462, 86.8% men and 70, 13.2% women, age range 12 to 80) with the following distribution: Pfizer-BioNTech: 367 (69%), Moderna: 137 (25.8%), AstraZeneca: 12 (2.3%), Janssen/Johnson & Johnson: 6 (1.1%), COVAXIN: 1 (0.1%), and unknown mRNA vaccine: 9 (1.7%). The distribution of patients receiving vaccine dosage was investigated. On cardiac MR Imaging, late intravenous gadolinium enhancement (LGE) was observed mainly in the epicardial/subepicardial segments (90.8%, 318 of 350 enhancing segments), with the dominance of inferolateral segment and inferior walls. Pericardial effusion was reported in 13.1% of cases. The vast majority of patients (94%, 500 of 532) were discharged from the hospital except for 4 (0.7%) cases. Post-COVID-19 myocarditis was most commonly reported in symptomatic men after the second or third dose, with CMRI findings including LGE in 90.8% of inferior and inferolateral epicardial/subepicardial segments. Most cases were self-limited.

## 1. Introduction

To mitigate the COVID-19 pandemic, mass vaccination of the general population commenced in December 2020 across the developed world. As of 7 March 2022, more than 60% of the world’s population has received at least one dose of several vaccines [1,2,3,4]. Widespread vaccination and other measures have resulted in decreased rates of COVID-19 incidence, severity, morbidity, and mortality [5]. However, vaccine-related side effects have been reported, including pain, redness, swelling or lymphadenopathy at or around the injection site, fever, fatigue, headache, muscle pain, nausea, vomiting, itching, chills, joint pain, and anaphylactic shock [4,6]. Recently, myocarditis has also been reported following the COVID-19 vaccination [7,8,9] with findings of impaired cardiac function detected clinically on electrocardiogram, echocardiogram, chest radiograph (CXR), contrast-enhanced chest computed tomography (CCT), contrast-enhanced coronary computed tomography angiography (CCTA), and gadolinium-enhanced cardiac magnetic resonance imaging (CMRI) [10,11,12]. The purpose of this study is to review the reported imaging findings of myocarditis after COVID-19 vaccination, stratified by vaccine type and imaging findings.

## 2. Methods

### 2.1. Search Strategy

We reviewed the literature initially on 23 July 2021, with an updated review on 10 March 2022, to identify all imaging studies reporting myocarditis after vaccination with any United States Food and Drug Administration (FDA) or World Health Organization (WHO) approved COVID-19 vaccine worldwide. All English language databases, including PubMed (MEDLINE), Scopus, Web of Science, Embase (Elsevier), and Google Scholar, were searched. The keywords search and medical subject headings (MeSH) were used: ‘Coronavirus’, ‘COVID-19’, ‘SARS-CoV-19’, ‘COVID-19 vaccination’, ‘Vaccine*’ ‘Post vaccine’, ‘Vaccination side effect’, and ‘Myocarditis’. The search strategy of the Medline database is presented in an Appendix A.

### 2.2. Eligibility Criteria

All reported English language studies reported cardiac imaging findings of patients who received one or more doses of FDA- or WHO-approved types of COVID-19 vaccines, including Pfizer-BioNTech, Moderna, AstraZeneca, Janssen/Johnson & Johnson, and COVAXIN vaccine; with the clinical, laboratory, and at least one imaging presentation of myocarditis by different imaging modalities such as CXR, echocardiography, CCT scan, CCTA, and CMRI, were included in the study. Conference abstracts, duplicate studies, reviews, and non-English languages were excluded from the study.

### 2.3. Data Extraction and Synthesis

**Figure 1:**
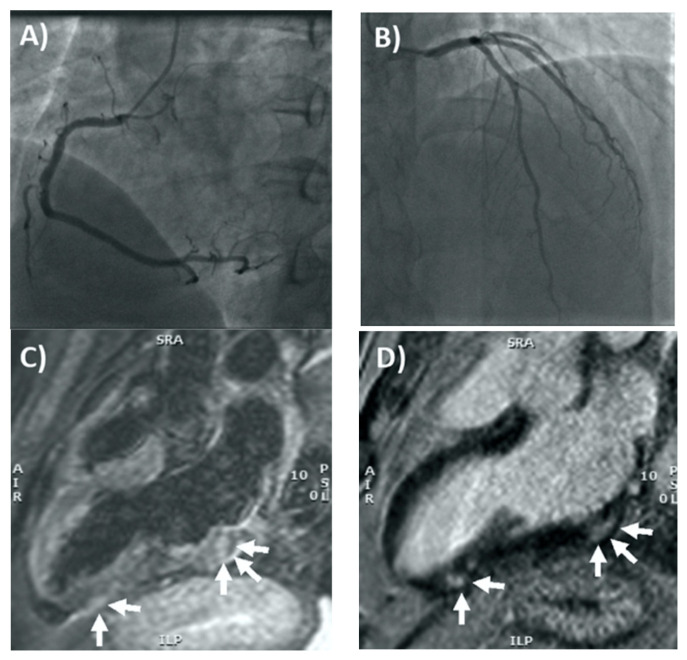
Coronary angiography and cardiac MRI. (**A**) The right coronary artery only had a mild plaque (<30% luminal diameter) in the mid portion, whereas in (**B**) the left main stem, left anterior descending artery, and circumflex artery had no evidence of coronary plaques. (**C**) T2-weighted 3-chamber view on cardiac MRI, showing focal areas of edema involving the subepicardial-intramyocardial regions of the basal and apical segments of the inferolateral wall (arrows). (**D**) Late gadolinium enhancement confirmed the presence of non-ischemic myocardial lesions in the basal and apical segments of the inferolateral wall (arrows) consistent with acute myocarditis. Reprinted/adapted with permission from Ref. [13] 2021, Elsevier.

**Figure 2:**
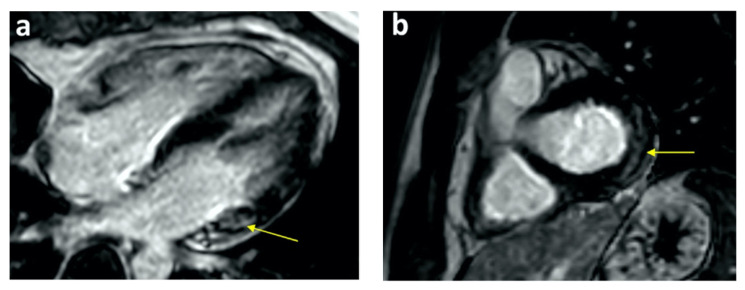
Cardiac MRI PSIR-LGE views show late gadolinium subepicardial enhancement in basal lateral segments in (**a**) four-chamber and (**b**) short-axis views. Reprinted/adapted with permission from Ref. [14]. 2021, Elsevier.

**Figure 3:**
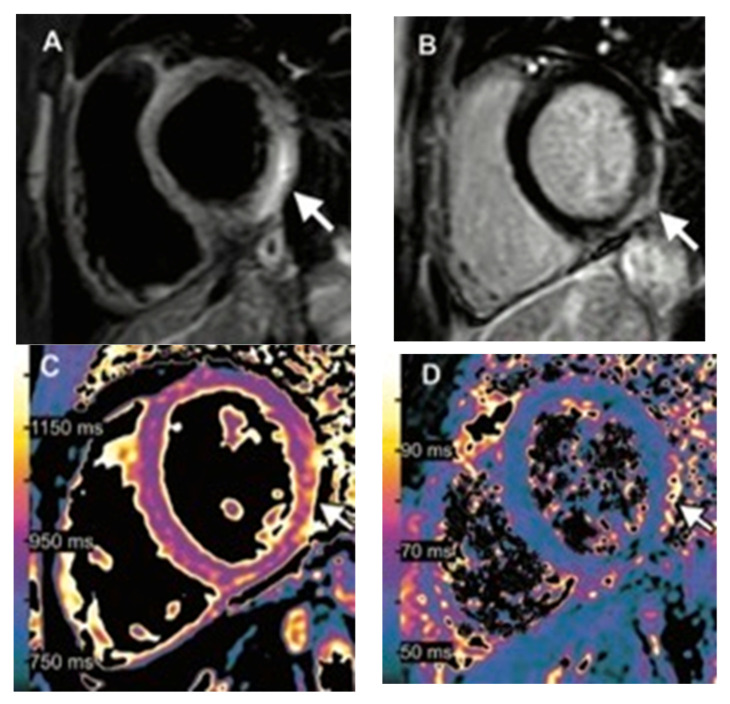
Images of a 15-year-old boy with myocarditis after COVID-19 vaccination. One day after receiving his second vaccination dose, he developed fever, myalgia, and intermittent tachycardia. (**A**) T2-weighted short inversion time inversion recovery MRI scans at 1.5 T in short-axis view show focal high-signal intensities (arrow) at the basal lateral and inferior wall, indicating myocardial edema. (**B**) Late gadolinium enhancement image in short-axis view shows corresponding linear subepicardial enhancement (arrow), indicating inflammatory myocardial necrosis. (**C**) T1 mapping and (**D**) T2 mapping in the short-axis view show elevated T1 and T2 at the mid-ventricular lateral and inferolateral wall (arrow in (**C**,**D**)), indicating acute myocardial injury (focal T1, 1165 ms; focal T2, 70 ms; institution-specific cut-off values for acute myocarditis: T1 global ≥ 1000 ms, T2 global ≥ 55.9 ms). Reprinted/adapted with permission from Ref. [8], 2021, RSNA.

**Figure 4:**
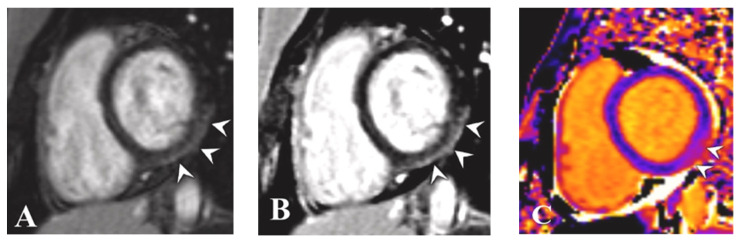

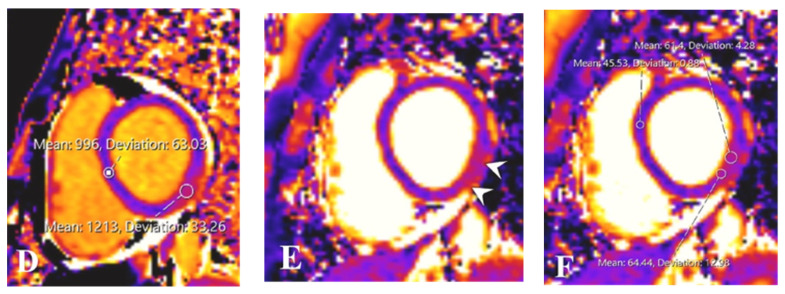
Magnetic Resonance Imaging of Case 2. Post-contrast magnitude inversion recovery (MAG-IR) (**A**) and phase-sensitive inversion recovery (PSIR) (**B**) images in short-axis views show subepicardial enhancement in the inferolateral wall at the base (arrowheads). Native T1 map shows corresponding abnormality (arrowheads in (**C**)) with elevated values (**D**) in the inferolateral wall compared with the interventricular septum. T2 mapping also showed abnormality in this region (arrows in (**E**)) with elevated values (**F**) when compared with the interventricular septum. Reprinted/adapted with permission from Ref. [15] 2021, Elsevier.

Two radiologists independently extracted the following data from the included studies and then cross-checked the results. A third reviewer (radiologist with 23 years of experience) resolved disagreements via consensus. The following data were extracted: the name of the first author, study’s region, study design, patient’s characteristic, type of COVID-19 vaccine, number of doses, the clinical and laboratory findings of myocarditis following vaccination, electrocardiographic (ECG) findings, echocardiographic findings, imaging findings (echocardiography, cardiac CT, CCTA, CMRI), and patient outcome. Meanwhile, most of the CMR findings in included studies were fulfilled based on the revised Lake Louise Criteria (LLC), including the appearance of at least one T1 (increased myocardial T1 relaxation times, extracellular volume fraction, or LGE) and T2-based criterion (increased myocardial T2 relaxation times, visible myocardial edema, or increased T2 signal intensity ratio) Additionally, we reported images of four vaccinated cases [8,13,14,15] presenting with myocarditis with abnormal cardiac MRI findings following COVID-19 vaccination after obtaining formal permission (Figure 1, Figure 2, Figure 3 and Figure 4).

## 3. Results

### 3.1. Literature Search

**Figure 5:**
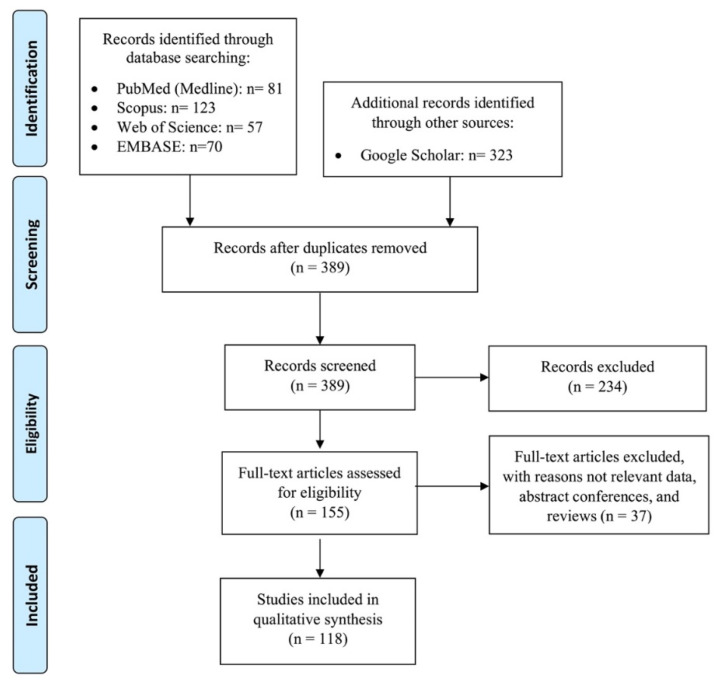
Flow diagram of the study selection process. Preferred reporting items for systematic reviews and meta-analyses (PRISMA). Adapted from Moher et al.

From the initial literature review, a total of 654 studies were derived and after removing duplicates, title and abstract screening were performed on 389 remaining studies. Finally, 118 studies were selected and identified as candidates for investigation based on our inclusion criteria. The flow diagram of the study selection process is presented in Figure 5.

### 3.2. Patients’ Characteristics

We derived 532 post-vaccine cases of CT or MR findings of myocarditis, including 462 (86.8%) males and 70 (13.2%) females (age range: 12–80 years). In total, 113 (21.2%) cases had positive disease history or comorbidities, presented in Table 1.

### 3.3. Clinical and Laboratory Findings

The most common reported symptoms were chest discomfort or substernal/positional chest pain (*n* = 429, 80.6%) and fever with or without chills in 245 (46%) cases. The clinical presentation/onset of symptoms after vaccination was not reported in 167 (31.4%) cases. In the study cohort, most cases (69%, 367 of 532 cases) injected Pfizer-BioNTech, followed by Moderna in 137 (25.8%) cases. In total, 62 (11.6%) cases received the first vaccine dose, 333 (62.6%) cases received two vaccine doses, and 7 cases received the third (booster) dose (Table 1 and Supplementary Table S1).

### 3.4. Presentation Date

The interval between vaccination date and symptoms was reported in 501 patients from 108 studies, of which 97 reported exact dates and 9 reported a range of days in the presentation interval. Meanwhile, the median interval of presentation days after vaccination in Pfizer-BioNTech, Moderna, AstraZeneca, Janssen/Johnson & Johnson, COVAXIN, and unknown mRNA vaccines were 3 (0–90), 3 (0–46), 6 (1–19), 4 (2–5), 8 (0–8), and 2 (0–20) days, respectively, in 297 cases (from 97 of 118 studies). In addition, eleven studies comprising 204 patients reported a median range of presentation days between 1 and 96 days. In almost all studies, concurrent COVID-19 infection was investigated by polymerase chain reaction (PCR) and antibodies lab findings of cases, resulting in negative results. Most patients were discharged from the hospital uneventfully. There were three reported deaths and one readmission following the post-COVID-19 vaccination myocarditis event. In other cases, the outcome was not reported (Table 1). The first reported death was a 42-year-old male who presented two weeks after the second dose of Moderna vaccine with an ejection fraction of 15% on echocardiography, without coronary artery disease (CAD), and died three days following hospitalization due to cardiogenic collision. On post-mortem, histological examination inflammatory exudates confirmed the myocarditis diagnosis. The second reported death was a 70-year-old female with multiple sclerosis, hospitalized two days after the Janssen/Johnson & Johnson vaccine with an ejection fraction (10%) and diffuse left ventricular hypokinesis. She died eight days after admission due to cardiogenic shock and renal failure. The third reported death was in a 62-year-old woman with melanoma who received the Janssen/Johnson & Johnson vaccine four days before presentation. The patient died after numerous advanced cardiovascular life support attempts due to a critically depressed ejection fraction.

### 3.5. Imaging Findings

In the 532 cases, imaging findings were reported using different imaging methods. Chest radiography (CXR) findings were reported in 43 of 532 (8.1%) cases, of which 6 out of 43 reported abnormalities, including pulmonary edema, cardiomegaly, congestion, and pleural effusion.

#### 3.5.1. CMRI and CCTA

The principal diagnostic imaging method of post-COVID-19 vaccine-related myocarditis reported in all 532 cases was CMRI, with abnormal findings in 361 (67.8%) cases. Abnormal findings included myocardial edema 188 (35.3%), patchy or global myocardial signal hyperintensity in T1-weighted 142 (26.7%) and T2-weighted images 150 (28.2%), and pericardial, epicardial, and subepicardial LGE (overall 234, 44%), which all suggest a diagnosis of myocarditis. LGE of cardiac MRI was observed mainly on the epicardial/subepicardial segments (318 of 350 locations of enhancement, 90.8%) with the involvement of the inferior and inferolateral walls. Moreover, septal involvement was scanty. (Table 2)

#### 3.5.2. Echocardiography

Echocardiography was reported in 73% (388 of 532) of cases with normal findings in 228 cases. Abnormal echocardiographic findings in 41.2% (160 of 388) of cases included pericardial effusion 5.1% (20 of 388), focal and general hypokinesia 12.1% (47 of 388), reduction in mono or biventricular ejection fraction 21.9% (85 of 388), and others were not reported. The left ventricle ejection fraction (LVEF) was reported in 197 cases of which 32% (63 of 197) were less than 50% and 68% (134 of 197) cases were greater than 50% (Supplementary Table S2).

## 4. Discussion

The COVID-19 vaccines have successfully mitigated the pandemic and decreased the transmission and severity of the disease, with few reported side effects such as myocarditis [16,17]. In this literature review, most patients with COVID-19 vaccine myocarditis were symptomatic men who presented mainly after the second or booster vaccine dose of mRNA vaccines and recovered rapidly. However, there are other cases with non-mRNA vaccines, unlike the last two studies [18,19] On CMRI, LGE reported mainly on the epicardial/subepicardial segments (90.8%) with the involvement of the inferior and inferolateral walls. Pericardial effusion was reported in the area of enhancement in around 13% of cases.

The incidence of post-vaccine myocarditis has been reported with many different vaccines, especially the smallpox vaccine [20,21,22,23]. Following the first report of post-COVID-19 vaccination myocarditis from the Israeli Ministry of Health, other reports were published [24,25].

The United States Centers for Disease Control (CDC) estimated that the risk of myocarditis among hospitalized patients was more than 15 times higher for patients with COVID-19 than those without COVID-19 infection [26]. Although the hallmark of myocarditis is inflammation in the myocytes on a myocardial biopsy, this is invasive, morbid and requires expertise. Non-invasive serum inflammatory factors and cardiac markers, ECG, and echocardiography modalities may be useful in the diagnosis of myocarditis [12,23,27]. CMRI, with its ability to resolve separate myocardial tissues and their composition, is considered the best non-invasive test due to its high specificity and sensitivity compared with other imaging modalities [28].

The diagnostic imaging criteria of myocarditis on CMRI include the appearance of at least one T1- and T2-based criterion according to the revised LLC [29,30]. In the present review, most included studies described LLC criteria such as myocardial signal hyperintensity and LGE as the dominant imaging findings in their cases. However, in an earlier study by Shiyovich et al., CMR imaging results were not compatible with the updated Lake Louise criteria for the early diagnosis of myocarditis; most of our reviewed cases had one or more updated LLC criteria that may fulfill them [19].

The earliest sign of myocarditis is myocardial edema associated with underlying inflammation. This finding can be detected mainly as T2 hyper signal areas in the myocardium; blood flow increases in inflamed tissue, which can be seen as an early enhancement [31]. These two early CMRI imaging findings are seen before other modalities could show signs of myocarditis, mostly changes in anatomical findings requiring more time (e.g., increased myocardial thickness or decreased ejection fraction in echocardiography and cardiomegaly, and increased vascularity in chest X-ray) [32].

Another CMRI finding which could be evaluated is the time and severity of the pathology (acute or subacute phase), as delayed enhancement is seen in fibrotic and necrotic changes. The main drawback of CMRI is its limited availability to secondary or tertiary centers mainly in the developed world. Imaging and non-imaging findings of other modalities, which are nonspecific signs of acute heart failure, should be evaluated in a clinical scenario in post-COVID-19 vaccinated patients presenting with symptoms.

Albert et al. [33] reported that a previously healthy 24-year-old man presented with chest pain that had worsened several days following his second dose of the Moderna COVID-19 vaccine. In workup, besides elevated serum inflammatory factors and troponin I, CMRI myocarditis findings meeting LLC included patchy mid-myocardial and epicardial LGE, normal LV size, and EF (58%), with superimposed edema. Rosner et al. [34] reported several different post-COVID-19 vaccine myocarditis cases.

One of the hypotheses about this appearance, due to intensified awareness of multi-system inflammatory syndrome in children (MIS-C) manifesting with indefinite symptoms, is more frequently assessed with a widespread panel of tests that enhance the sensitivity of identifying episodes of perimyocarditis that might not need intervention [35,36]. The above-mentioned literature presented vaccines’ diversity and prevalence. All imaging findings in various vaccinated cases by different types of vaccine, which complicated with myocarditis, were alike without significant variation. In addition, this review study indicates that this adverse event has happened mainly in adolescents and youthful adults with male gender type.

### Limitations

This presented review study has some limitations. First, most included studies did not report the number and proportion of patients with a prior history of COVID-19 infection. Second, some studies did not separately report the second and booster vaccine shots, so we cannot consider separating the second from the booster dose under vaccination status.

## 5. Conclusions

In most reported cases of post-COVID-19 vaccination-related myocarditis on CMRI, LGE was observed mainly in the epicardial/subepicardial segments, with the involvement of inferolateral and inferior walls with pericardial effusion in the area of enhancement in 13.1% of cases.

## Figures and Tables

**Table 1:** Characteristics of Cases with Myocarditis Following COVID-19 Vaccination (*n* = 532).

Characteristics	Number (%)
**Gender**	
male	462 (86.8)
female	70 (13.2)
**Age** (range, y/o)	12–80
**Vaccine type**	
Pfizer-BioNTech	367 (69)
Moderna	137 (25.8)
Janssen/Johnson & Johnson	6 (1.1)
AstraZeneca	12 (2.3)
COVAXIN	1 (0.1)
unknown	9 (1.7)
**Vaccine status**	
first dosage	62 (11.6)
second dosage	333 (62.6)
booster dosage	7 (1.4)
unknown	130 (24.4)
**Patients’ presentation**	
chest pain	429 (80.6)
fever/chills	245 (46)
myalgia	132 (24.8)
headache	87 (16.3)
shortness of breath	161 (30.2)
fatigue	71 (13.3)
nausea/vomiting	65 (12.2)
**Comorbidities**	
hypertension	34 (6.4)
coronary artery disease	10 (1.9)
autoimmune disease	17 (3.2)
myocarditis history	7(1.3)
others *	45 (8.4)
**Outcome**	
discharged	500 (94)
readmitted	1 (0.1)
expired	3 (0.6)
unknown	28 (5.3)

Each **bold** words are the name of a characteristic. * Other comorbidities include cancer, type 2 diabetes, asthma, chronic obstructive pulmonary disease (COPD), chronic kidney disease (CKD), Von Willebrand disease, anxiety disorder, and Lennox–Gastaut syndrome.

**Table 2:** Cardiac MRI Findings of Study Cases based on the vaccine’s type.

Vaccine Type	Location of Enhancement (Based on Cardiac MRI Reported)	
	Epicardial/Subepicardial	Pericardial
Pfizer-BioNTech	232	20
Moderna	70	9
Janssen/Johnson & Johnson	2	0
AstraZeneca	8	3
Unknown type	6	0
Total	318	32

**Noted.** Some of the cases had multiple locations of enhancement.

## Supplementary Materials

The following are available online at https://www.mdpi.com/article/10.3390/tomography8040164/s1, Table S1: Characteristics of Cases with Myocarditis Following COVID-19 Vaccination (*n* = 532), Table S2: Cardiac Findings of Cases with Myocarditis Following COVID-19 Vaccination (*n* = 532). References [7,8,9,13,14,15,16,19,33,34,37,38,39,40,41,42,43,44,45,46,47,48,49,50,51,52,53,54,55,56,57,58,59,60,61,62,63,64,65,66,67,68,69,70,71,72,73,74,75,76,77,78,79,80,81,82,83,84,85,86,87,88,89,90,91,92,93,94,95,96,97,98,99,100,101,102,103,104,105,106,107,108,109,110,111,112,113,114,115,116,117,118,119,120,121,122,123,124,125,126,127,128,129,130,131,132,133,134,135,136,137,138,139,140,141,142,143,144] are cited in the supplementary materials.

## Abbreviations

CMRI: Cardiac magnetic resonance imaging, CCTA: Coronary computed tomography angiography, CAD: Coronary artery disease, LGE: Late gadolinium enhancement, LVEF: Left ventricle ejection fraction, CXR: Chest radiography, LLC: Lake Louise Criteria.

## Appendix A

Keyword Search Strategy
Medline (PubMed)
(“coronavirus 2” [Title/Abstract] OR “coronavirus 2” [Mesh] OR “coronavirus infections” [Title/Abstract] OR “coronavirus infections” [Mesh] OR “COVID-19” [Title/Abstract] OR “COVID-19” [Mesh] OR “coronavirus” [Title/Abstract] OR “coronavirus” [Mesh] OR “2019-nCoV” [Title/Abstract] OR “COVID-2019” [Title/Abstract] OR “COVID19” [Title/Abstract] OR “nCoV” [Title/Abstract] OR “coronavirus disease 2019” [Title/Abstract] OR “2019 novel coronavirus” [Title/Abstract] OR “severe acute respiratory syndrome” [Title/Abstract] OR “SARS-CoV-19” [Title/Abstract] OR “SARS-CoV-2” [Title/Abstract] OR “2019-CoV-19” [Title/Abstract] OR “SARS-CoV” [Title/Abstract] OR “2019nCoV” [Title/Abstract] OR “coronavirinae” [Title/Abstract] OR “2019 Novel Coronavirus Infection” [Title/Abstract] OR “2019 nCoV Infection” [Title/Abstract] OR “Bat coronavirus” [Title/Abstract] OR “betacoronavirus*” [Title/Abstract] OR “coronavirus Infection Disease 2019” [Title/Abstract] OR “COVID*” [Title/Abstract] OR “Novel Coronavirus Pneumonia” [Title/Abstract] OR “Wuhan virus” [Title/Abstract]) AND (“Vaccination” [Title/Abstract] OR “Vaccination” [Mesh] OR “Vaccines” [Title/Abstract] OR “Vaccines” [Mesh] OR “Mass Vaccination” [Title/Abstract] OR “Mass Vaccination” [Mesh] OR “Immunization” [Title/Abstract] OR “Immunization” [Mesh] OR “Immunization Programs” [Title/Abstract] OR “Immunization Programs” [Mesh] OR “Vaccination*” [Title/Abstract] OR “Vaccin*” [Title/Abstract] OR “Vaccination program” [Title/Abstract] OR “Vaccine” [Title/Abstract] OR “Vaccine-mediated protection” [Title/Abstract] OR “Post-vaccine” [Title/Abstract] OR “Post vaccine” [Title/Abstract] OR “Vaccination-induced” [Title/Abstract] OR “Post-vaccination” [Title/Abstract] OR “Post vaccination” [Title/Abstract] OR “Immunization*” [Title/Abstract] OR “Pfizer vaccine” [Title/Abstract] OR “Pfizer-BioNTech COVID-19 vaccine” [Title/Abstract] OR “Moderna vaccine” [Title/Abstract] OR “mRNA vaccine” [Title/Abstract] OR “Astra-Zeneca vaccine” [Title/Abstract] OR “anti-SARS-CoV-2 vaccine” [Title/Abstract] OR “BNT162b2 mRNA COVID-19 vaccine” [Title/Abstract]) AND (“Myocarditis” [Title/Abstract] OR “Myocarditis” [Mesh] OR “Myopericarditis” [Title/Abstract] OR “Cardiomayopathy” [Title/Abstract] OR “Pricarditis” [Title/Abstract] OR “Myocardial lesion” [Title/Abstract] OR “acute myocarditis” [Title/Abstract] OR “myocard*” [Title/Abstract] OR “Dilated Cardiomyopathy” [Title/Abstract])
