# Supplementary material for: Myocarditis Following COVID-19 Vaccination: Cardiac Imaging Findings in 118 Studies
Source: Tomography. 2022 Jul 30;8(4):1959–73. doi: 10.3390/tomography8040164 (PMC9416085; doi:10.3390/tomography8040164)
Supplement: Supplementary file 1 [file tomography-08-00164-s001.zip › tomography-1777867-supplementary.pdf]

Supplementary TABLE S1. Characteristics of Cases with Myocarditis Following COVID-19 Vaccination (n = 532)

First author (Ref)	Study design	Patient's characteristics	Vaccine type (dosage)	Patient's presentation	Outcome
Albert, USA (1)	Case report(n=1)	Male (24y/o)	Moderna(2nd)	Chest pain (sub-sternal), fever/chills, and body aches	Discharged
Ammirati, Italy (2)	Correspondence (n=1)	Male(56y/o)	Pfizer-BioNTech(2nd)	Chest pain (acute onset)	Discharged
D'Angelo, Italy (3)	Case report (n=1)	Male(30y/o)	Pfizer-BioNTech(2nd)	Chest pain (retrosternal), dyspnea,nausea, sweating,fever,arthralgia	Discharged
Deb, USA (4)	Case report (n=1)	Male(67y/o) *	Moderna(2nd)	SOB, fever/chills, nausea, orthopnea, fatigue	Discharged
Garcı´a, Spain (5)	Scientific letter (n=1)	Male (39 y/o) #	Pfizer-BioNTech(2nd)	Chest pain, inter-scapular pain, fever	Discharged
Habib, Qatar (6)	Case report (n=1)	Male (37y/o), ex-smoker, drinking	Pfizer-BioNTech(2nd)	Chest pain (retrosternal), generalized body aches, fever/chills, headache	Discharged
Shaw, USA (7)	Imagine vignette(n=4)	2 Male (16-24 y/o)<br>2 Female (17-31 y/o)	Pfizer-BioNTech(1st): 1<br>Pfizer-BioNTech(2nd): 2<br>Moderna(1st): 1	Chest pain	NR
Watkins, USA (8)	Case report (n=1)	Male (20y/o), tobacco abuser	Pfizer-BioNTech(2nd)	Chest pain (mid-sternal), SOB	Discharged
Singh B, USA (9)	Case report (n=1)	Male (24y/o)alcohol socially	Pfizer-BioNTech(2nd)	Chest pain, headache, fever/chills, fatigue	Discharged
Muthukumar, USA (10)	Case report (n=1)	Male (52y/o), HLP, OSA, hepatic steatosis	Moderna(2nd)	Chest pain (sub-sternal), fever/chills,myalgia, headache	Discharged
Minocha, USA (11)	Brief report (n=1)	Male (17y/o)	Pfizer-BioNTech(2nd)	Chest pain, fever, body aches	Discharged
McLean, USA (12)	Case report (n=1)	Male (16y/o)	Pfizer-BioNTech(2nd)	Chest pain (sharp/stabbing)	Discharged
Dickey, USA (13)	Case series(n=6)	6 Male (16-40y/o)	Pfizer-BioNTech(2nd): 5	Chest pain, fever/chills,myalgia,headache,neck pain,rhinorrhea,malaises	Discharged

			Moderna(2nd): 1		
Kim HW, USA (14)	Brief report (n=4)	3 Male (23- 36 y/o)<br>1 Female (70y/o), HLP, cigarette smoking	Moderna(2nd): 2<br>Pfizer-BioNTech(2nd): 2	Chest pain,SOB, injection site discomfort, fatigue/muscle ache, fever/chills, headache, diaphoresis, syncope, palpitations	Discharged
Larson, Italy (15)	Case series (n=8)	8 Male (21-56 y/o)	Moderna(2nd): 3<br>Pfizer-BioNTech(2nd): 5	Chest pain, fever/chills,myalgia, SOB,cough	Discharged
Vidula, USA (16)	Case series (n=2)	2 Male(18-19y/o)	Pfizer-BioNTech(2nd): 1<br>Moderna(2nd): 1	Chest pain (sub-sternal), SOB,fevers, myalgia	Discharged
Mansour, USA (17)	Cardiothoracic imaging (n=2)	1 Male (25 y/o)<br>1 Female (21 y/o)	Moderna(2nd): 2	Chest pain (sub-sternal), fever/chills	Discharged
Marshall, USA (18)	Case series (n=7)	7 Male(14-19y/o)	Pfizer-BioNTech(2nd): 7	Chest pain,fever,SOB, fatigue, bilateral arm pain,nausea,vomiting,anorexia, myalgia,abdominal pain,palpitation,headache, weakness	Discharged
Montgomery, USA (19)	Brief report (n=23)	23 Male	Pfizer-BioNTech(1st): 1<br>Pfizer-BioNTech(2nd): 6<br>Moderna(1st): 2<br>Moderna(2nd): 14	Chest pain	Discharged
Mouch, Israel (20)	Short communication (n=6)	6 Male(17-45 y/o)	Pfizer-BioNTech(1st): 1<br>Pfizer-BioNTech(2nd): 5	Chest pain	Discharged
Nevet, Israel (21)	Brief communication (n=3)	3 Male(20-29 y/o)	Pfizer-BioNTech(2nd): 3	Chest pain, fever	NR
Rosner, USA (22)	Case series (n=7)	7 Male(19-39 y/o)	Pfizer-BioNTech(2nd): 5<br>Moderna(2nd): 1<br>Johnson & Johnson: 1	Chest pain,SOB, fever/chills,myalgia,headache	Discharged

Schauer, USA (23)	Case series (n=13)	12 Male(12-17 y/o)<br>1 Female (12-17 y/o)	Pfizer-BioNTech(2nd): 13	Chest pain, fever/chills, SOB, myalgia, headache, malaise, vomiting	Discharged
Park, USA (24)	Brief report (n=2)	2 Male (15-16 y/o)	Pfizer-BioNTech(1st): 1<br>Pfizer-BioNTech(2nd): 1	Chest pain,fever, headache	Discharged
Cereda, Italy (25)	Case report (n=1)	Male(21y/o)	Pfizer-BioNTech (2nd)	Chest pain, fever, myalgia	Discharged
Williams, USA (26)	Case report (n=1)	Male (34y/o)	Moderna(2nd)	Chest pain (pleuritic retrosternal), fever	Discharged
Starekova, USA (27)	Research letter (n=5)	4 Male(17-38 y/o)<br>1 female (32 y/o)	Pfizer-BioNTech(2nd): 3<br>Moderna(2nd): 2	Chest pain, fever/chills, malaise, headache, dyspnea, nausea, lightheadedness,body ache,fatigue	Discharged
Nassar, USA (28)	Case report (n=1)	Female (70 y/o), multiple sclerosis	Janssen/Johnson& Johnson	Dyspnea	Expired
Di Tano, Italy (29)	Case report (n=1)	Male(29y/o)	Moderna(1st)	Fever, precordial and inter-scapular pain	Discharged
Khogali, Qatar (30)	Case report (n=1)	Female (29 y/o)	Moderna (2nd)	Fever, fatigue	Discharged
Tano, USA (31)	Brief report (n=8)	8 Male (15-17 y/o)	Pfizer-BioNTech (1st): 2<br>Pfizer-BioNTech (2nd): 6	Chest pain	Discharged
Issak, Germany (32)	Images in radiology (n=1)	Male (15 y/o)	Pfizer-BioNTech (2nd)	Fever, myalgia	Discharged
Verma, USA (33)	Correspondence (n=2)	1 Male (42 y/o)<br>1 Female (45 y/o)	Pfizer-BioNTech(1st): 1<br>Moderna(2nd): 1	Chest pain, dyspnea, dizziness	Discharges/ Expired
Diaz, USA (34)	Researchletter (n=57)	42 Male, 15 Female	Pfizer-BioNTech: 32<br>Moderna: 23<br>Janssen/Johnson&Johnson: 2	NR	Discharged
Levin, Israel (35)	Short communication	7 Male (18-24 y/o)	Pfizer-BioNTech (2nd): 7	Chest pain, fever, fatigue	Discharged

	(n=7)				
Shumkova, Bulgaria (36)	Clinical vignette (n=1)	Male (23 y/o)	Pfizer-BioNTech (2nd)	Chest pain, fever, SOB	Discharged
Cimaglia, Italy (37)	Case report (n=1)	Male (24 y/o)	Pfizer-BioNTech (2nd)	Radiating chest pain worsened by breathing	Discharged
Dionne, USA (38)	Case series (n=15)	14 Male<br>1 Female (12-18 y/o)	Pfizer-BioNTech(1st): 1<br>Pfizer-BioNTech(2nd): 14	Chest pain,fever, myalgia, headache	Discharged
Ehrlich, Germany (39)	Letter to Editor (n=1)	Male (40 y/o)	Pfizer-BioNTech(1st)	Chest pain, SOB, headache	Discharged
Gautam, USA (40)	Case report (n=1)	Male (66 y/o)	Pfizer-BioNTech (2nd)	Crushing substernal chest discomfort, diaphoresis, emesis	Discharged
Kim IC, Korea (41)	Case report (n=1)	Male (24 y/o)	Pfizer-BioNTech (2nd)	Chest pain(sub-sternal)	Discharged
Jain, USA (42)	Case series (n=63)	58 Male, 5 Female<br>(12-20 y/o)	Pfizer-BioNTech: 59<br>Moderna: 4	Chest pain, fever, SOB, nausea	Discharged
King, USA (43)	Short communication (n=4)	3 Male (20-30 y/o)<br>1 Female (23 y/o)	Pfizer-BioNTech (2nd): 1<br>Moderna(2nd): 3	Chest pain	Discharged
Matta, USA (44)	Case report (n=1)	Male (27 y/o)	Pfizer-BioNTech (2nd)	Chest pain (central, sharp), fatigue	Discharged
Patel, USA (45)	Case series (n=5)	5 Male (19-37 y/o)	Pfizer-BioNTech(1st): 1<br>Pfizer-BioNTech (2nd): 3<br>Moderna(2nd): 1	Fever/chills, nausea	Discharged
Patrignani, Italy (46)	Case report (n=1)	Male (56 y/o)	Pfizer-BioNTech(1st)	Epigastric pain, tachycardia, hypotension	Discharged
Sulemankhil, USA (47)	Case report (n=1)	Male (33 y/o)	Janssen/Johnson&Johnson	Chest pain (sub-sternal) myalgia, fever/chills	Discharged
Tailor, USA (48)	Case report (n=1)	Male (44 y/o)	Moderna(2nd)	Chest pain (severe), dyspnea, headache	Discharged
Ujueta, USA (49)	Case report (n=1)	Female (62 y/o)	Janssen/Johnson&Johnson	Weakness, body aches (progressive), worsening	Expired

				fatigue	
Hung, Taiwan (50)	Case report (n=1)	Male (23 y/o)	AstraZeneca (1st)	Fever, myalgia, sore throat	Discharged
Kaneta, Japan (51)	Case report (n=1)	Male (25 y/o)	Moderna (2nd)	Fever	Discharged
Kaul, USA (52)	Case report (n=2)	2 Male (21 and 28 y/o)	Pfizer-BioNTech (2nd): 1<br>Moderna (2nd): 1	Chest pain (sub-sternal), fever/chills, headache	Discharged
Kim D, Korea (53)	Case report (n=1)	Male (29 y/o)	Pfizer-BioNTech (2nd)	Chest pain	Discharged
Koizumi, Japan (54)	Case report (n=2)	2 Male (22 and 27 y/o)	Moderna (2nd): 2	Chest pain	Discharged
Maki, Japan (55)	Image Focus (n=1)	Female (20 y/o), case of Kawasaki disease	Moderna (2nd)	Chest pain, dyspnea, fever	Discharged
Miqdad, SAU (56)	Case report (n=1)	Male (18 y/o)	Pfizer-BioNTech (2nd)	Chest pain (sub-sternal)	Discharged
Nguyen, Germany (57)	Case report (n=1)	Male (20 y/o)	Moderna (1st)	Chest pain (mid-sternal), myalgia, fatigue, fever	Discharged
Onderko, USA (58)	Case report (n=3)	3 Male (25, 28, 36 y/o)	Pfizer-BioNTech (2nd): 2<br>Moderna (2nd): 1	Chest pain, myalgia	Discharged
Pareek, USA (59)	Case series (n=11)	10 Male, 1 Female (16-53 y/o)	Pfizer-BioNTech (2nd): 9<br>Moderna (2nd): 1<br>Astrazenca: 1	Chest pain (sub-sternal), fatigue, headache, myalgia	Discharged
Perez, USA (60)	Case series (n=7)	6 Male, 1 Female (22-71 y/o)	Pfizer-BioNTech (2nd): 3<br>Moderna (2nd): 4	Chest pain, dyspnea, fatigue	Discharged
Sakaguchi, Japan (61)	Case report (n=1)	Male (49 y/o)	Pfizer-BioNTech (2nd)	Fever, cough, orthopnea	Discharged
Schmitt, France (62)	Brief reports (n=1)	Male (19 y/o)	Pfizer-BioNTech (2nd)	Chest pain	Discharged
Sivakumaran, UK (63)	Image Focus (n=1)	Male (20 y/o)	Pfizer-BioNTech (2nd)	Chest pain	Discharged
Viskin, Israel (64)	Research letter (n=8)	8 Male (20-34 y/o)	Pfizer-BioNTech (2nd): 8	Chest pain, malaise	Discharged

Vollmann, Germany (65)	Clinical snapshot (n=1)	Male (28 y/o)	Pfizer-BioNTech (NR)	Chest pain, fever, fatigue	Discharged
Wilson, USA (66)	Cardiothoracic Imaging (n=1)	Female (16 y/o)	Pfizer-BioNTech (2nd)	Chest pain	Discharged
Shiyovich, Israel (67)	Retrospective study (n=15)	15 Male (22.5-40 y/o)	Pfizer-BioNTech (2nd): 15	Chest pain, fever	Discharged (14)<br>Readmitted (1)
Hasnie, USA (68)	Case report (n=1)	Male (22 y/o)	Moderna (1st)	Chest pain	Discharged
Habedank, Germany (69)	Letter to the editors (n=1)	Male (60 y/o)	Moderna (2nd)	Fever, dizziness, fainting	Discharged
Goenawan, USA (70)	Case report (n=1)	Male (67 y/o), Diabetes, HLP, CKD	Pfizer-BioNTech (2nd)	Chest pain (sub-sternal)	Discharged
Fleming-Nouri, USA (71)	Research letter (n=8)	8 Male (16-24 y/o)	Pfizer-BioNTech (2nd): 8	Chest pain	Discharged
Das, USA (72)	Retrospective case series (n=25)	22 Male, 3 Female (12-18 y/o)	Pfizer-BioNTech (1st): 3<br>Pfizer-BioNTech (2nd): 22	Chest pain, fever/chills, SOB, headache, vomiting, nausea, myalgia	Discharged
Chen, UK (73)	Case report (n=1)	Male (16 y/o)	Pfizer-BioNTech (1st)	Chest pain, fever, myalgia	Discharged
Chelala, USA (74)	Retrospective study (n=5)	5 Male (16-19 y/o)	Pfizer-BioNTech (2nd): 4<br>Moderna (2nd): 1	NR	Discharged
Boursier, France (75)	Image of the month (n=2)	2 Male (18 and 21 y/o)	Moderna (2nd): 1<br>Pfizer-BioNTech (2nd): 1	NR	NR
Badshah, USA (76)	Case report (n=1)	Female (22 y/o)	Moderna (2nd)	Chest pain (sub-sternal), SOB, fever/chills, myalgias	Discharged
Azir, USA (77)	Clinical communications (n=1)	Male (17 y/o)	Pfizer-BioNTech (2nd)	Chest pain (sub-sternal), dyspnea	Discharged
Ambati, USA (78)	Case report (n=2)	2 Male (16 and 17 y/o)[$]	Pfizer-BioNTech (2nd): 2	Chest pain (sub-sternal)	Discharged
Sokolska, Poland (79)	Clinical vignette (n=1)	Male (21 y/o)	Pfizer-BioNTech (1st)	Chest pain	NR

Lazaros, Greece (80)	Short communication (n=8)	3 Male, 5 Female (46-76 y/o)	Pfizer-BioNTech (1st): 2, (2nd): 3, Moderna (2nd): 1 AstraZeneca (1st): 2	Chest pain, dyspnea, fever	Discharged
Facetti, Italy (81)	Case report (n=1)	Male (20 y/o)	Pfizer-BioNTech (2nd)	Chest pain, headache, fever, diffuse malaise	Discharged
Meyer-Szary, Poland (82)	Case series (n=3)	3 Male (12, 17, 29 y/o)	Pfizer-BioNTech (2nd): 2<br>Moderna (2nd): 1	Chest pain, dyspnea, fever	NR
Aikawa, Japan (83)	Case report (n=1)	Male (37 y/o)	Moderna (1st)	Chest pain (acute)	Discharged
Bricoli, Italy (84)	Case series (n=4)	4 Male	mRNA vaccine (2nd): 4	Chest pain	NR
Chachar, Bahrain (85)	Case report (n=1)	Male (24 y/o)	Pfizer-BioNTech (1st)	Chest pain	Discharged
Eggebrecht, Germany (86)	Letter to the editor (n=4)	2 Male, 2 Female (14-46.9 y/o)	Pfizer-BioNTech (NR): 2, Moderna (NR): 2	Chest pain, SOB, palpitation, fatigue	NR
Istampoulouoglou, Switzerland (87)	Case series (n=17)	12 Male, 5 Female (17-88 y/o)	Pfizer-BioNTech (1st): 3, (2nd): 2 Moderna (2nd): 8, (1st):3, Unknown (2nd): 1	Chest pain, fever/chills, palpitation, fatigue	Discharged
Lim, South Korea (88)	Case report (n=1)	Female (38 y/o)	Pfizer-BioNTech (NR)	Chest pain	Discharged
McCullough USA (89)	Case report (n=1)	Male (23 y/o)	Moderna (2nd)	Chest pain, fever/chills	Discharged
Murakami, Japan (90)	Case series (n=2)	2 Male (27, 37 y/o)	Pfizer-BioNTech (2nd): 1 Pfizer-BioNTech (1st): 1	Chest pain	Discharged
Nagasaka Japan (91)	Case report (n=1)	Male (23 y/o)	Pfizer-BioNTech (2nd)	Chest pain, fever	Discharged
Parmer, USA (92)	Case series (n=4)	3 Male, 1 Female (19-53 y/o)	mRNA (2nd): 3, mRNA (1st): 1	Chest pain	Discharged
Singh R, India (93)	Case series (n=2)	1 Male (52 y/o), 1 Female (65 y/o)	AstraZeneca (1st): 1, AstraZeneca (2nd): 1	Chest pain, fever, dyspnea, cough	NR

Takeda, Japan (94)	Letter to the editor (n=1)	1 Male (53 y/o)	Pfizer-BioNTech (2nd)	Chest pain, dyspnea	NR
Tinoco, Portugal (95)	Case report (n=1)	1 Male (39 y/o)	Pfizer-BioNTech (2nd)	Chest pain, fever, headache	Discharged
Tiwari, India (96)	Case report (n=1)	1 Male (33 y/o)	COVAXIN (1st)	Eyes swelling, itching, SOB	NR
Di Dedda, Italy (97)	Case series (n=27)	25 Male,2 Female<br>(12-80 y/o)<br>3Auto immune disease	AstraZeneca (1st): 1<br>AstraZeneca (2nd): 2,<br>Pfizer-BioNTech(1st): 8<br>Pfizer-BioNTech (2nd): 10<br>Moderna (1st):3<br>Moderna (2nd):3	Chest pain, palpitations, myalgia, dyspnea, fever, arthralgia	Discharged (25)<br>NR (2)
Fronza, Canada (98)	Case series (n=21)	17 Male,4 Female<br>(mean:31y/o ± 14)<br>Smoking	Moderna :12<br>Pfizer-BioNTech: 9<br>Second dose: 17	Chest pain, palpitations, SOB	Discharged
Bews, Canada (99)	Case series (n=10)	9 Male (2 trans from female to male), 1 Female<br>(18-45y/o)<br>2 IBS, 2 Depression, 1 ADHD	Pfizer-BioNTech (2nd): 3<br>Moderna (1st):1<br>Moderna (2nd):6	Chest pain (pleuritic)	Discharged
Manfredi , Italy (100)	Case series (n=6)	4 Male , 2 Female<br>(14-25 y/o)<br>AVNRT,RBBB	Pfizer-BioNTech (2nd):4<br>Moderna (2nd):2	Fever	Discharged
Sharff, U.S. (101)	Case series (n=6)	4 Male , 2 Female<br>(18-39 y/o)	Pfizer-BioNTech(booster):5<br>Pfizer-BioNTech (2nd):1	Chest pain, cardiogenic shock	Discharged

Nunn, Germany (102)	Case series (n=4)	3 Male , 1 Female<br>(16-47 y/o)<br>Sjogren syndrome, myocarditis	Pfizer-BioNTech(1st):1<br>Pfizer-BioNTech (2nd):2<br>Moderna (2nd):1	Chest pain, fever, flu-like syndrome, dyspnea, back pain, headache, sore throat , cough	Discharged
Bengel, Germany (103)	Case series (n=2)	2 Male (20 y/o , 23y/o)	Moderna (2nd): 2	General malaise and fatigue, chest discomfort, arthralgia, fever, epigastric pain	Discharged
Ohnishi, Japan (104)	Case report (n=1)	Male , 26y/o	Pfizer-BioNTech (2nd)	Fever, headache, appetite loss, general malaise, shoulder stiffness	Discharged
Owuor , Kenya (105)	Case report (n=1)	Female , 36 y/o	AstraZeneca (1st)	Chest pain(stabbing & crushing) , fatigue, palpitations, dyspnea, breathlessness, fever, headache, muscle aches	Discharged
Sano, Japan (106)	Case report (n=1)	Male, 20 y/o	Moderna (2nd)	Chest pain, fever	Discharged
Wong, Australia (107)	Case report (n=1)	Male,20 y/o<br>Asthma ,depression	Pfizer-BioNTech (2nd)	Chest pain (pleuritic), fever , diaphoresis	Discharged
Wu, USA (108)	Case report (n=1)	Male,40 y/o	Pfizer-BioNTech(1st)	Chest pain (pleuritic) ,dyspnea , nausea, myalgia , fever	Discharged
Yen, Taiwan (109)	Case report (n=1)	Male,32y/o<br>Gouty arthritis, dyslipidemia	Moderna (1st)	Breathlessness, local reaction(pain,swelling),loose stool , dyspnea,chest discomfort(substernal)	Discharged
Mohammadi ,Iran (110)	Case report (n=1)	Male, 20y/o<br>Multiple sclerosis	AstraZeneca(3rd):booster	Chest pain (retrosternal)	Discharged
Lee, South Korea (111)	Case report (n=1)	Male , 22y/o	Moderna (2nd)	Chest pain	NR
Kyaw , USA (112)	Case report (n=1)	Male , 24y/o	Pfizer-BioNTech (2nd)	Chest pain (midsternal) and pressure, palpitations, myalgias, chills	Discharged
Kounis , Greece	Case report (n=1)	Male, 21y/o	Pfizer-BioNTech(1st)	Chest discomfort	Discharged

(113)		Asthma, Allergy			
Kerkhove, Belgium (114)	Case report (n=1)	Male , 50 y/o<br>Iinsulin-dependent diabetes mellitus	AstraZeneca (2nd)	Fever, malaise ,SOB , chest pain	Discharged
Kawakami, Japan (115)	Case report (n=1)	Female , 45 y/o	Moderna (2nd)	Chest pain , fever	Discharged
Gill, USA (116)	Case report (n=1)	Male, 44 y/o	Moderna (2nd)	Chest pain, SOB	Discharged
Agdamag, USA (117)	Case report (n=1)	Female , 80 y/o<br>Aappendectomy, cholecystectomy, hysterectomy	Pfizer-BioNTech (1st)	Emesis , generalized abdominal discomfort, diarrhea , chest pain (atypical)	Discharged
Fosch, Spain (118)	Case report (n=1)	Male,24 y/o<br>IBD (Crohn)	Pfizer-BioNTech (3nd): booster	Chest pain , fever	Discharged

Note.SOB: Shortness of breath, **HLP:**Hyperlipidemia,**OSA:** Obstructive sleep apnea, **HTN:** Hypertension, **CKD:** Chronic kidney disease,**AVNRT**:Atrioventricular nodal reentrant tachycardia, RBBB: Right bundle branch block, **IBD** : inflammatory bowel disease **, NR:** Not reported

*This case had history of HTN, Type 2 diabetes mellitus, Congestive heart failure, CABG, Chronic obstructive pulmonary disease (COPD), Gastro-esophageal reflux disease (GERD), hypothyroidism, and HLP

#This case had history of asthma, autoimmune hypothyroidism, chronic atrophic gastritis, an isolated episode of AF, and recurrent spontaneous pneumothorax

$Case 1 had a history of Von-Willebrand disease, anxiety disorder, and Lennox-Gastaut syndrome; case 2 had a history of asthma

Supplementary Table S2. Cardiac Findings of Cases with Myocarditis Following COVID-19 Vaccination (n = 532)

First author (ref)	ECG findings	Imaging findings
Albert, USA (1)	Sinus rhythm	CMRI: Enhancement (mid-myocardial, epicardial, patchy), superimposed edema, CCTA: Normal, Echo: LVEF: 65%

Ammirati, Italy (2)	Sinus rhythm, ST elevation (precordial), peaked T waves	CMRI: Enhancement (subepicardial-intramyocardial, focal), edema (basal and apical segments of the inferolateral wall), CCTA: Normal<br>CXR: Normal (n=1), Echo: NR
D'Angelo, Italy (3)	ST elevation, T waves change	CMRI: Enhancement (subepicardial), myocardial and pericardial hyperintensity signal, pericardium thickness, T1: diffuse myocardial late gadolinium enhancementwith subepicardial distribution and sparing of the basal and mid septal segments;thickening and enhancement of pericardium can also be seen/ T2: increased subepicardial signal intensity of the inferolateralmyocardial segments (arrows). Increased thickness and signal intensity of pericardium isalso shown. CCTA: Normal, Echo: Pericardial effusion
Deb, USA (4)	Sinus tachycardia, ST segment and T wave changes	CXR: Normal, Echo: LVEF: 50-54%, Dilated Lt. atriumHypokinesia, Diastolic dysfunction (grade II)
Garci'a, Spain (5)	Sinus tachycardia, ST elevation	CMRI: Enhancement (subepicardial, lateral mediastinal), edema, CCTA: Normal<br>CXR: Normal, Echo: Normal
Habib, Qatar (6)	ST elevation (ant. and inf. leads)	CMRI: Enhancement (subepicardial, basal lat.), CCTA: Normal, Echo: LVEF: 57%
Shaw, USA (7)	ST elevation (ant. lead)	CMRI: Edema (n=4), including epicardial (n=3) and mid wall (n=1), Epicardial fibrosis (n=3), non-ischemic myocardial injury (n=4), T1: nonischemic myocardial injury, Echo: NR
Watkins, USA (8)	ST elevation, PR depressions<br>(V5, V6, II, aVF)	CMRI: Myocarditis, Cardiac CT: Normal,Bed side cardiac ultrasound: Pericardial effusion<br>CCTA: NR, Echo: LVEF: 59%
Singh, USA (9)	Sinus rhythm, ST depression(lead III)	CMRI: Enhancement (subepicardial, lat. wall of the Lt. ventricle), CCTA: Normal, CXR: Normal, Echo: EF:55%
Muthukumar, USA (10)	Sinus rhythm, Rt. bundle branch block	CMRI: Enhancement (mid myocardial and subepicardial, linear and nodular, in the inferoseptal, inferolateral, anterolateral, and apical walls), T1: elevated T1 relaxation time and relative inhomogeneity. CCTA: Normal, Echo: Normal
Minocha, USA (11)	ST elevation (diffuse)	CMRI: Enhancement (subepicardial, mid-lat., apical), pericardial effusion, Echo: PVC
McLean, USA (12)	Sinus rhythm, ST elevation (diffuse, V2-V6, I and aVL leads)	CMRI: Myocardial fibrosis, myocardial hyperemia, pericardial effusion<br>CCTA: Enhancement (subepicardial, lat. wall), Echo: EF: 61%

Dickey, USA (13)	Ectopic atrial rhythm (n=1), ST elevation (n=5), PR depression (n=2), T wave abnormality (n=1)	CMRI: Enhancement (patchy mid myocardial &subepicardial), CCTA: NR, Echo: LVEF ≥ 50% (n=3), LVEF < 50% (n=3)
Kim, USA (14)	ST elevation (n=4), PR depression (n=2)	CMRI: Enhancement (n=4) (apical lat., Multiple, lat.), regional wall motion abnormality (n=4), pericardial effusion (n=4), (epicardial (n=3), myocardial (n=1), Coronary angiography: Normal (n=1), Chest CT: Normal (n=2), CXR: Normal (n=4)
Larson, Italy (15)	ST elevation (n=6), ST depression (n=3)<br>T wave peaked (n=1), Another (normal)	CMRI: Delayed enhancement (n=8), including subepicardial (n=2), myocardial (n=1)), edema (n=6): (pericardial (n=1)), pericardial effusion (n=3), Echo: LVEF ≥ 50% (n=6), LVEF< 50% (n=2), Hypokinesis (n=8)
Vidula, USA (16)	ST elevation (diffuse) (n=2)	CMRI: Enhancement (subepicardial (n=2)), T1: regional<br>elevation in the lateral wall (n=2)<br>Coronary angiography: Normal (n=1)<br>CCTA: Normal (n=1), Echo: EF: 47% (n=1)<br>EF: 59% (n=1)
Mansour, USA (17)	ST elevation (n=2)<br>PR depression (n=1)	CMRI: Enhancement (subepicardial, mid Lat. wall) (n=2), increased T1 and T2signal intensity<br>Cardiac CT: Normal (n=1), Echo: LVEF ≥ 50% (n=2)
Marshall, USA (18)	AV dissociation and junctional, escape rhythm (n=1)<br>ST elevation (n=6)<br>T wave abnormality (n=2)<br>Sinus bradycardia(n=1)<br>Low voltage extremity leads (n=1)	CMRI: Enhancement (subepicardial (n=3), epicardial (n=1))<br>Myocardial edema (n=6)<br>Axillary adenopathy (n=1)<br>Elevated extracellular volume fraction (n=1)<br>Fibrosis (n=1), hyperemia (n=2), MR (n=1), T1: early post- contrast heterogeneity indicating hyperemia, late gadolinium enhancement, myocardial fibrosis, in the subepicardial mid and apical left ventricle free wall (n=1). Echo: Borderline basal Lat. and Post. strain (n=1)<br>LVEF< 50% (n=1), Normal (n=5)

Montgomery, USA (19)	ST elevations, T-wave inversions (n=19)<br>Others normal (n=4)	CMRI: Enhancement (subepicardial) and/or focal edema (myocardial) (n=8)<br>CMRI: NR (n=15)<br>Coronary artery imaging: cardiac catheterization (n=11), CCTA (n=5)<br>Normal (n=16), NR (n=7), Echo: LVEF< 50% (n=4)<br>LVEF ≥ 50% (n=19)
Mouch, Israel (20)	Sinus tachycardia (n=1)<br>ST elevation (n=6)<br>T inversion (n=2)<br>PR depression (n=1)<br>ST depression (n=1)<br>S_I Q_{III} T_{III} (n=1)	CMRI: Enhancement (subepicardial (n=2), myocardial (n=3)), Echo: Hypokinesis (n=1)<br>Edema: (myocardial (n=4), subepicardial (n=2))<br>CCTA: Normal (n=1)<br>Coronary angiography: Normal (n=1), Echo: Normal (n=5)
Nevet, Israel (21)	ST elevation (diffused) (n=3)	CMRI: Enhancement (myocardium), inflammatory necrosis. myocardial edema (n=3), Echo: Normal (n=3)
Rosner, USA (22)	Sinus tachycardia (n=2)<br>ST elevation (n=2)<br>Nonspecific ST-T change (n=1)<br>PR depression (n=1)<br>T wave inversion V1(n=1)<br>Others normal (n=2)	CMRI: T1 & T2: Enhancement (subepicardial (n=6), mid myocardial (n=3), pericardium (n=2), antroseptal mid wall (n=1))<br>Myocardial edema (n=3)<br>Coronary angiography: NR (n=4), no CAD (n=3), coronary stenosis (n=1) (30% proximal circumflex)<br>CXR: Normal (n=7), Echo: LVEF ≥ 50% (n=6)<br>LVEF< 50% (n=1), Hypokinesis (n=3)
Schauer, USA (23)	ST elevation (n=7)<br>Non-specific T wave changes (n=1)<br>T wave inversion (n=1)<br>Others normal (n=4)	CMRI: Enhancement (patchy subepicardial to transmural) (n=13)<br>Edema (n=13)<br>LV wall motion abnormalities (n=2)<br>Focal hypokinesis LV (n=2), EF ≥ 55% (n=11)

		EF< 55% (n=2)
Park, USA (24)	ST elevation (n=2)<br>T wave inversion (n=1)	CMRI: Enhancement (early myocardium) (n=1), NR (n=1), T1: mild global early enhancementof the myocardium, with pronounced enhancement in the subendocardial layer of the leftventricle<br>CXR: Normal (n=2), Echo: LVEF (60-63%) (n=2)<br>Patchy myocardium echogenicity (n=1)
Cereda, Italy (25)	ST elevation (diffuse)<br>T inversion (inferolateral)	CMRI: Enhancement (epicardial, non-ischemic patchy pattern)<br>Myocardial edema (patchy, ant., inf., lat.), mild pericardial effusion<br>Coronary angiography: Normal<br>CXR: Normal (n=1), Echo: LV hypokinesis (inf., post.)
Williams, USA (26)	PR depression (Lat.)<br>ST elevation (mirrored in aVR)<br>PR elevation, ST depression	CMRI: Enhancement (subepicardial and pericardial, anterolateral, inferolateral)<br>Patchy myocardial edema, Echo: EF: 43%
Starekova, USA (27)	ST elevation (n=2)<br>ST depression (n=1)<br>T-wave abnormal (n=3)<br>RSR' pattern V1 (n=1)	CMRI: Enhancement (epicardial (n=5), myocardial (n=2), pericardial (n=5))<br>Pericardial effusion (n=3)<br>Ipsilateral axillary lymphadenopathy (n=4)<br>CCTA: Normal (n=3)
Nassar, USA (28)	Sinus tachycardia<br>T wave inversion (V4-V6 leads)	Echo: AR, LV Hypokinesis<br>LVEF:10%, Reduced contraction (apex/distal ant.)
Di Tano, Italy (29)	ST elevation (Inf and Lat leads)<br>Negative T waves (precordial leads)	CMRI: Enhancement (subepicardial, inf. apical wall)<br>Myocardial edema (apex)<br>CXR: Normal, Echo: Normal
Khogali, Qatar (30)	Sinus tachycardia<br>ST elevation, Short PR interval	LVEF:27%<br>Pericardial effusion

	Abnormal T wave	Cardiac tamponade
Tano, USA (31)	ST elevation (n=4)<br>ST depression (n=2)<br>PR depression (n=1)<br>Abnormal T waves (n=1)<br>Intraventricular conduction delay (n=1)<br>PVC (n=1)	CMRI: Enhancement (n=3)<br>Myocardial edema (n=3)<br>CMRI: NR (n=5)<br>CXR: Normal (n=8), Echo: EF ≥ 50% (n=8)<br>MVP (n=1)<br>MR (n=1)<br>Foramen oval (n=1)<br>Aortic root dilation (n=1)
Issak, Germany (32)	ST elevation (precordial leads)	CMRI: Enhancement (subepicardial)Myocardial edema (lat. and inferolateral walls)Pericardial effusion, inflammatory necrosis, and acute myocardial infarction, elevated T1 and T2 relaxation times at the mid ventricularlateral and inferolateral wall, indicating acute myocardial injury. Echo: Normal
Verma, USA (33)	ST depression (n=1)<br>ST elevation (diffuse) (n=1)	CMRI: NR<br>Coronary angiography: Normal (n=2), Echo: LVEF:15-20% (n=2)<br>LVH (n=1)
Diaz, USA (34)	Bundle branch block (n=3)<br>ST elevation (n=23)<br>PR depression (n=7)	Echo: LVEF <50% (n=8)
Levin, Israel (35)	Tachycardia (n=1)<br>ST elevation (n=5)<br>ST depression (n=2)<br>PR elevation (n=1)<br>PR depression (n=2)	CMRI: Enhancement (subepicardial (n=3), mesocardiac (n=1), myocardium (n=2), basal LV (n=2), septum (n=1))<br>Myocardial edema (n=1)<br>CMRI: NR (n=4)<br>Cardiac CT: Enhancement (subepicardial, lat. and Inf. walls) (n=1)

	LVH criteria (n=1)<br>PVC (n=1)	Late adherence (lat., inf., apex, septum) (n=1)<br>Cardiac angiography: Normal (n=1),<br>CXR: Normal (n=3), Echo: LVEF ≥ 50% (n=5)<br>LVEF < 50% (n=2)<br>Hypokinesis (apex) (n=1)<br>Pericardial effusion (n=1)
Shumkova, Bulgaria (36)	Sinus rhythm<br>ST elevation (inf., V4-V6 leads)	CMRI: Interstitial edema (subepicardial distribution LV walls (apical, lat., inf.))<br>Coronary angiography: Normal, Echo: LVEF: 45%<br>Hypokinesis (ant., lat., inf. wall)<br>Bull eye map: LV impaired longitudinal strain<br>(ant. lat./apical)
Cimaglia, Italy (37)	Sinus tachycardia<br>ST elevation (Inf. leads)<br>ST depression (V1-V3 leads)	CMRI: Enhancement (patchy, non-ischemic subepicardial distribution), myocardial hypersignal intensity in the inf., inferolateral, and anterolateral walls<br>Coronary angiography: Normal, Echo: EF: 45%<br>Inf-post. wall hypokinesia, hyper echogenicity of the pericardium
Dionne, USA (38)	ST elevation (diffuse) (n=9)	CMRI: Enhancement (n=12), regional hyperintensity (n=2), elevated LV global native T1 (n= 2)
Ehrlich, Germany (39)	Sinus rhythm	CMRI: Enhancement on the fat saturated LGE (focal to diffuse), moderate and diffuse high signal of the Lt. ventricleMyocardial edema, Echo: Median EF: 58.6<br>(Range, 43.7 to 64.7)
Gautam, USA (40)	ST elevation (Ant. leads)	CMRI: Enhancement (myocardial and epicardial) along the ant. septum in the mid-ventricular level (LVEF: 44%)Coronary angiography: Normal<br>CXR: Normal
Kim IC, Korea (41)	ST elevation (mild)<br>(I, II, aVF, and V2-6 leads)	CMRI: T1 (n=4) & T2 (n=3): Hyper signal intensity (subepicardial pattern of LGE in basal inf. and inferolateral segments), Echo: Normal myocardial contractilityPericardial effusion (mild)

Jain, USA (42)	ST elevations (diffuse) and/or T wave inversion (n=44)<br><br>Complete heart block (n=1)	CMRI: 56 pts,<br><br>T2: Myocardial edema (n=50)<br><br>LGE (subepicardial, inferolateral and lat. walls of the Lt. ventricle) (n=49), T1: evaluate EGE denoting hyperemia (n=13), universally negative. evidence of LGE (n=49), in the inferolateral and lateral walls of the left ventricle in the sub-epicardial region. Echo: LVEF 45-54% (n=9) , Bicuspid aortic valve (n=1) anomalous origin of the Rt. coronary artery from the Lt. sinus of Valsalva (n=1)
King, USA (43)	Down-sloping PR depressions<br><br>ST elevation (diffuse) (n=3)<br><br>T-wave inversions, Lat. leads (n=1)	CMRI: Enhancement (subepicardial, inf., basal to mid inferolateral, mid anterolateral, apical lat., apical septal, and apical inf. wall segments) (n=1), T1: diffusely elevated T1 relaxationtimes, most prominently in the lateral and septal wall segments<br><br>CMRI: NR (n=3), Echo: LVEF 45-60% (n=3)<br><br>LVEF 60-70% (n=1)<br><br>Hypokinesis (n=2)<br><br>Normal (n=2)
Matta, USA (44)	Sinus rhythm	CXR: Normal, Echo: NR
Patel, USA (45)	PR depression (diffuse) and PR elevation (aVR lead) (n=3)<br><br>Sinus tachycardia (n=1)<br><br>ST elevation (Lat. leads), ST depression (V1 lead) (n=1)	CMRI: Enhancement (subepicardial, mid-myocardial; basal, mid, and apical lat segments) (n=5)<br><br>Myocardial edema (n=4), T1: Late gadolinium enhancement, Echo: LVEF: 50-65%<br><br>Normal (n=5)
Patrignani, Italy (46)	Sinus rhythm	CMRI: Enhancement (subepicardial, non-ischemic lesions in the basal and middle segments of the inferolateral wall)<br><br>Edema: (focal, intramyocardial regions of the ant. wall and inferolateral wall)<br><br>Coronary angiography: Normal<br><br>CXR: Normal, Echo: Normal
Sulemankhil, USA	Sinus rhythm	CMRI: Enhancement (focal area of myocardium in the mid to apical lat. region of the Lt.

(47)		ventricle), Echo: NR
Tailor, USA (48)	ST elevation<br>(Lat. limb and precordial leads)	CMRI: Enhancement (patchy, linear, mid-myocardial, septum and inf. walls of mid-ventricle, subepicardial/mid-myocardial, lat. wall at the mid-ventricle), myocardial edema<br>Coronary angiography: Mild CAD<br>CXR: Mild pulmonary edema, Echo: Borderline enlarged Lt. ventricle EF: 40-45%<br>Lt. ventricular hypokinesis<br>Rt. Enlargement of ventricle with decreased systolic function
Ujueta, USA (49)	Sinus tachycardia<br>T wave inversions, septal leads<br>(with Rt. atrial enlargement)	Coronary angiography: cardiac output: 5.1 L/min, cardiac index: 3.26 L/min/m2, LVEDP: 37 mmHg<br>CXR: Normal, Echo: Severe biventricular cardiomyopathy<br>(LVEF: 29%)<br>Mild pericardial effusion
Hung, Taiwan (50)	Sinus tachycardia, T wave inversion change	CMRI: Enhancement (endocardial and epicardial), and in pleural effusion<br>CXR: Bilateral pleural effusion, pleuralcentesis,<br>Echo: Anteroseptalhypokinesia with pericardial effusion
Kaneta, Japan (51)	ST elevation (inferior and lateral leads)	CMRI: Enhancement (posterolateral segment)<br>Coronary angiography: Posterolateralhypokinesia, Echo: Inferolateralhypokinesia
Kaul, USA (52)	ST elevation (inferolateral/diffuse)	CMRI: Enhancement (epicardial (lateral), pericardial and epicardial surface (anterior and lateral segments)), Coronary angiography: Normal, Echo: LVEF: 55% with inferolateralhypokinesia, LVEF: 25% with dilated RV (mild)
Kim D, Korea (53)	Normal sinus rhythm	CMRI: Scanty pericardial effusion without specific findings<br>CXR: Cardiomegaly (mild), Echo: LVEF: 61.7% with mild pericardial effusion
Koizumi, Japan (54)	Case 1 1: ST elevation (I, III, aVF, V3 to V6 leads), Case 2: ST elevation (II, III, aVF leads)	CMRI: Enhancement (epicardial (mid-wall and inferolateral segment of Lt. ventricle), T1: elevated native T1 values and ECVin the regions with LGE (n=1).

		Coronary angiography: Normal in both cases<br><br>Echo: Normal echocardiography (both cases)
Maki, Japan (55)	ST elevation (II, V1-V4 leads), sinus tachycardia	CMRI: Enhancement (Rt. ventricle of intraventricular septum, free wall of Rt. ventricle, pericardial), Coronary angiography: Normal<br><br>CXR: Significant cardiomegaly<br><br>Echo: Hypokinesia with mildly pericardial effusion
Miqdad, SAU (56)	ST elevation (I. II, V2-V6 leads)	CMRI: Enhancement (subpericardial) with Lt. ventricular myocardial edema, Coronary angiography: Normal, Echo: Normal with LVEF:63%
Nguyen, Germany (57)	Normal	CMRI: T1/T2: Enhancement (subepicardial (mid and basal inferolateral segments)<br><br>CXR: Normal<br><br>Echo: Normal
Onderko, USA (58)	Normal (case 1), ST elevation (cases 2 and 3)	CMRI: Enhancement (multiple focal in mid-wall) and myocardial edema (case 1), Enhancement (multiple focal in the basal inferior, mid to distal lateral/inferolateral walls)) and myocardial edema (cases 2 and 3),<br><br>Coronary angiography: Normal<br><br>Echo: Normal echo (all cases)
Pareek, USA (59)	ST elevation (6 cases)	CMRI reported in 9 cases: Enhancement (epicardial (inferolateral/anterior and lateral walls) in 6 cases, subepicardial (lateral basal wall/inferolateral) in 2 cases, pericardial in 2 cases), myocardial edema in 4 cases, T1: inhomogeneity with patchy areas of increased relaxation time (n=1). Echo: Normal in reported cases
Perez, USA (60)	ST segment changes (5 cases)	CMRI reported in 6 cases: Enhancement (pericardial in 3 cases), Echo: Decreased LVEF in 3 cases, Rt. ventricular dysfunction in 3 cases
Sakaguchi, Japan (61)	Decreased voltage in the limb leads	CMRI: Enhancement (diffuse and global) with myocardial edema, T1: increased globalnative T1 value, LVEF: 33%<br><br>CXR: Mildly congestion<br><br>Echo: NR

Schmitt, France (62)	ST elevation (persistent)	CMRI: Enhancement (subepicardial (lateral, middle to apical lateral of Lt. ventricle segments))) with myocardial edema<br><br>Echo: Normal with LVEF: 72%
Sivakumaran, UK (63)	ST elevation (persistent)	CMRI: Enhancement (subepicardial (inferior wall segment)), T1: elevated lateral LV myocardialT1.<br><br>Echo: LVEF: 50%
Viskin, Israel (64)	ST elevation (diffused, II, III, aVF leads) in 5 cases	CMRI reported in 7 cases: Enhancement (patchy, subepicardial and mid-myocardial, lateral wall) in 6 cases, myocardial edema in 1 cases, Echo: Decreased LVEF of 45-50% in 3 cases
Vollmann, Germany (65)	ST elevation	CMRI: Enhancement (subepicardial) with myocardial edema, Echo: NR
Wilson, USA (66)	NR	CMRI: Enhancement (subepicardial; basal inferolateral and inferior walls, mid-ventricular, antro/inferolateral, and apical lateral wall), Echo: NR
Shiyovich, Israel (67)	ST elevation (12 cases)	CMRI: T1/T2: Enhancement (mid-wall in 6 cases, epicardial in 5 cases, mid-wall and epicardial in 2 cases), pericardial effusion in 7 cases, T1: increased native values (n=6), Echo: NR
Hasnie, USA (68)	ST elevation (diffuse), PR depression (V3-V6 leads)	CMRI: Enhancement (subepicardial (lateral wall, inferior segments of mid-ventricular and apical of Lt. ventricle, adjacent pericardial)<br><br>Coronary angiography: Normal, Echo: Hypokinesia (anterolateral and mid-apical anterior segments), LVEF: 50-55%
Habedank, Germany (69)	Normal with sinus rhythm	CMRI: Enhancement (subepicardial (anterolateral medial) with myocardial edema, Echo: Normal
Goenawan, USA (70)	Ectopic atrial rhythm, Lt. ventricular hypertension, prolonged QTc	Chest CT: Cardiomegaly with bilateral pericardial effusion and pleural effusion, CXR: Bilateral pleural effusion and atelectasis, Echo: LVEF: 55-60%, tricuspid regurgitation (mild to moderate)
Fleming-Nouri, USA (71)	ST elevation (II, II, aVF, V3-V6 leads) in 7 cases	CMRI: Enhancement (epicardial (inferolateral) in 1 case, anterolateral Lt. ventricle in 1 case, sub-epicardial in 1 case), myocardial edema in 2 cases, fibrosis in 2 cases, Echo: Hypokinesia (inferoseptal) in 1 case, others were normal
Das, USA (72)	ST elevation in 15 cases, ST and PR depressions both in 1 case	CMRI reported in 16 cases: Enhancement (subpericardial (5 cases), sub-epicardial and mid-myocardial (6 cases), midmyocardial and subendocardial (1 case), mid-myocardial (1 case)), with myocardial edema in 6 cases, pericardial effusion in 3 cases, Echo: Normal in 23 cases, decreased

		LVEF from 48-49% in 2 cases
Chen, UK (73)	ST elevation (inferolateral leads), PR depression	CMRI: Enhancement (subepicardial (basal to mid-inferior, lateral segments), with myocardial edema and fibrosis, Echo: Lt. ventricular systolic dysfunction (LVEF: 48%)
Chelala, USA (74)	ST elevation (1 case), sinus bradycardia (1 case)	CMRI: Enhancement (subepicardial (4 cases), mid-to-subepicardial (1 case), with basilar, inferolateral, anterolateral, and inferior walls involvement, myocardial edema in 4 cases, pericardial effusion in 5 cases, Echo: Hypokinesia and decreased LVEF in 1 case
Boursier, France (75)	NR	DOTATOC-PET reported: Increased myocardial uptake (inferior/lateral walls) with myocardial/blood SUV max ratio of more than 2.2 (both cases), Echo: NR
Badshah, USA (76)	Sinus tachycardia	CMRI: Enhancement (pericardial, apical septal wall) with myocardial edema, decreased LVEF: 48% and hypokinesia, Chest CT: Small pleural effusion, atelectasis, Echo: NR
Azir, USA (77)	ST elevation (I, aVL leads), reciprocal depression (Lead III)	CMRI: Enhancement (subepicardial, anterior and lateral walls of Lt. ventricle), CCTA: Normal, CXR: Normal, Echo: NR
Ambati, USA (78)	ST elevations in both cases	CCTA: Normal, Echo: Decreased LVEF in 1 case
Sokolska, Poland (79)	ST elevation (II, III, aVF leads)	CMRI: Enhancement (diffuse subepicardial), with myocardial edema, CCTA: Normal, Echo: Normal with LVEF: 58%
Lazaros, Greece (80)	Normal	CMRI reported in 1 case: Enhancement (pericardial) with edema, Echo: Pericardial effusion: large in 4 cases, mild/moderate in 4 cases
Facetti, Italy (81)	ST elevation (aVR, V4-V6 leads)	CMRI: Enhancement (subepicardial (inferolateral basal segment)) with myocardial edema, CXR: Normal, Echo: Normal
Meyer-Szary, Poland (82)	ST elevation (I, II, V3 to V6 leads)	CMRI: Enhancement (subepicardial/intramural (inferolateral and basal segment)), with myocardial edema, T1: increasing relaxation time (n=2), Echo: Normal
Aikawa, Japan (83)	ST elevation (I, II, V3 to V6 leads)	CMRI: Enhancement (subepicardial), Echo: Abnormality in wall motion of LV
Bricoli, Italy (84)	ST elevation (diffuse)	CMRI: Enhancement (subepicardial), Echo: NR
Chachar, Bahrain (85)	ST elevation (V1-V6 leads)	CMRI: Enhancement (subepicardial, inferolateral, mid-lateral, and basal segments), Echo: Normal, LVEF: 55%
Eggebrecht, Germany (86)	ST elevation (n=1),ST depression (n=1)	CMRI: T1/T2: Enhancement (subepicardial), with myocardial edema, Echo: Pericardial effusions (n=2), with normal LVEF

Istampoulouoglou, Switzerland (87)	Abnormal in 10 cases	CMRI: T1/T2: Enhancement (subepicardial, inferolateral walls) (n=7), Echo: Abnormal in 6 cases
Lim, South Korea (88)	ST elevation (V1-V3 leads)	CMRI:T1/T2: Enhancement (multifocal and mid-wall), with myocardial edema (anterior and lateral segments), elevated T1 and T2relaxation times at the mid ventricular level. Echo: LV wall thickening, akinesia
McCullough, USA (89)	Normal	CTA: Normal, CMRI: NR, Echo: Septal wall thickening, abnormal motion, LVEF: 65%, pericardial effusion
Murakami, Japan (90)	ST elevation (V2-V6 leads)	CMRI: Enhancement (subepicardial, inferolateral, mid-basal, anterolateral segments), Echo: Pericardial effusion with septal wall thickening (9mm)
Nagasaka, Japan (91)	ST elevation (V3-V6 leads)	CMRI: Enhancement (epicardial, mid-myocardial in inferolateral), CXR: Plural effusion, cardiomegaly, Echo: Mild pericardial effusion, wall motion abnormality in anteroseptal
Parmer, USA (92)	ST elevation (diffuse)in 2 cases	CMRI: Enhancement (pericardial, inferolateral and lateral segments) in 2 cases, Echo: Normal
Singh R, India (93)	ST depression (V1-V6 leads), sinus tachycardia,	CMRI: Enhancement (subendocardial), Echo: LV systolic dysfunction, LVEF: 35-49%, hypokinesia, and pericardial effusion
Takeda, Japan (94)	NR	CMRI: Enhancement (mid ventricular septum and apex segments), Echo: Mild pericardial effusion and hypokinesia
Tinoco, Portugal (95)	ST elevation (V3-V6, I, II, aVL leads), and aVR ST depression	CMRI: Enhancement (subepicardial), Echo: Global hypokinesia, LVEF: 48%
Tiwari, India (96)	Sinus tachycardia	CMRI: Enhancement (LV myocardial) with full thickness, Echo: NR
Di Dedda, Italy (97)	ST elevation and T inversion in 21 cases	CMRI: Enhancement (non-ischemic mid-subepicardial in 23 cases, mid-epicardial in 18 cases, mid-wall in 3 cases, epicardial in 2 cases, pericardial in 1 case),
Fronza, Canada (98)	ST segment elevation(diffuse concave upward in aVR and V1),upright T wavesand PR depression	CMRI:Enhancement (Mid-wall in 4 cases, subepicardial in 13 cases, pericardial in 9 cases), pericardial edema in 4 cases, pericardial effusion in 4 cases ,high T1 in 14, high T2 in 16, LVEF: <55% (in 6 cases)
Bews, Canada (99)	STabnormalities (diffuse)	CMRI: Enhancement (sub-epicardial, mid-myocardia (inferior, inferolateral, lateral)), regional wall motion abnormalities in 1 case<br>Echo: LVEF:56%mild regional wall motion abnormalities (basal to mid inferior and inferolateral walls),GLS: reducedprimarily epicardial distribution in 4 cases

Manfredi, Italy (100)	Normal	CMRI: Myocardial edema , enhancement (inferolateral in 3 cases, pericardium in 2 cases, RV)<br>Echo: Pericardial effusions in female
Sharff, USA (101)	ST abnormality in 5 cases: ST elevation in 3 cases , diffuse ST changes in 1 cases<br>Sinus tachycardia	Echo: EF : 55-65% in 5 cases, 35-40 % in 1 case
Nunn, Germany (102)	Sinus rhythm, ST elevation in 2 cases	CMR: LGE (subepicardial scar, basal and midventricular pericardium)<br>Echo: LVEF:51%, abnormal wall motion (inferolateral)
Bengel, Germany (103)	Sinus rhythm, rSr0 configuration in V1–V3 anda slight notch in aVL ,ST-elevation in I, aVL, V4–V6, ST-depression in V1 and aVR.	CMR: Edema of the anterolateral myocardial segments, hypokinesis of theanterolateral myocardial segments, LEG (a non-ischaemic pattern lateral left ventricular wall, subepicardial, pericardium, non-ischaemic pattern of myocardial), pericardium thickening<br>Chest X-ray: Pulmonary congestion<br>Echo: Diffuse hypokinesis and an LVEF of 40%
Ohnishi, Japan (104)	ST elevation with upward concavity (I, II, aVL,aVF, V4 to V6), small Q wave (II, III, aVF)	CMRI: Edema ,LEG (a mid-myocardial and epicardial distribution in LV, subepicardial in anterior wall and interventricular septum near the apex)<br>Angiography: Normal<br>Thoracic CT: Normal<br>Echo : Normal
Owuor , Kenya (105)	first-degree atrioventricular (AV) block	CMRI: Patchy mid wall hyper intense areas, enhancement (mid septal)<br>Coronary angiogram: Normal<br>Chest x-ray: borderline cardiomegaly<br>Echo :Normal
Sano, Japan (106)	ST elevation (extensive), PR depression , T inversions	CMR: LVEF (38.3%) , hyperintense (anterolateral to inferolateral), hyperemia and edema (myocardial) , LEG (Sub-epicardial and mid-myocardial in the anterolateral to inferolateral)<br>Coronary angiogram: Normal

		Chest x-ray: Normal<br>Echo : LVEF: 42.4% ,inferolateral hypokinesis, abnormal wall motion
Wong, Australia (107)	Sinus tachycardia ,ST elevation (widespread)	CMRI: Hypokinesis of the mid and apical lateral , LEG: epicardial<br>Chest x-ray: Normal<br>Echo :Normal
Wu, USA (108)	Diffuse ST elevations , ST depressions (lead III , aVR ,V1) , PR depressions	CMRI: Myocardial edema, enhancement : inferior wall of the basal left ventricular myocardium<br>Coronary angiogram: Normal<br>Chest x-ray: Normal<br>Echo: Mild global hypokinesis , EF: 47%, pericardial effusion
Yen, Taiwan (109)	Sinus tachycardia	CMRI: LGE: Lateral wall subepicardium of LV, increased signal (small crescent area) , focal edema<br>Coronary angiogram: Normal<br>Echo : Biventricular function without visualized regional wall motion abnormality
Mohammadi ,Iran (110)	ST elevation (inferior limb, precordial leads)	CMRI: Myocardial (hyperemia ,edema in inferior and anterior apical) ,enhancement (basal inferior apical, anterior of LV)<br>Echo: Normal
Lee, South Korea (111)	NR	CMRI : LGE (basal posterior wall LV)
Kyaw , USA (112)	Sinus tachycardia	Coronary angiogram: Normal<br>Chest x-ray : Normal<br>CT-angiogram : Normal<br>Echo: Normal, EF : 55%
Kounis , Greece (113)	NR	CMRI: LGE (diffuse subepicardial) , increased signal T2 , increased values T1 & T2

		CT-angiogram: Normal
Kerkhove, Belgium (114)	Normal sinus rhythm	CMRI : Belated captation of contrast (inferolateral LV)<br><br>Echo :Normal
Kawakami, Japan (115)	T invert (inferior leads (II, III, and aVF))	CMRI: Edema (myocard) , LEG: diffuse hyperenhancement (apex, inferior , lateral wall)<br><br>Coronary angiogram: Normal<br><br>Chest x-ray : Mild pulmonary edema<br><br>Echo: EF (reduced) , abnormal wall motion (apex , inferior, lateral)
Gill, USA (116)	ST elevations (the inferior leads (II, III, aVF))	CMRI: Mid anterolateral and inferolateral: Edema (LV), enhancement (LV)<br><br>Coronary angiogram: Normal<br><br>Echo :Normal
Agdamag, USA (117)	Sinus tachycardia, non-specific ST and T changes, diffuse low voltage QRS complexes	CMRI : Diffuse myocardial edema , diffuse mid-myocardial fibrosis ,LEG : diffuse patchy mid-myocardial<br><br>Abdominal & pelvic CT : Anasarca<br><br>Echo: LV hypertrophy, LVEF: 20% - 35%, hypokinesis
Fosch, Spanish (118)	ST elevation (diffuse ,compatible concave), PR elevation aVR	CMRI: Edema, LGE : subepicardial and patchy location<br><br>Echo: EF: 56%

Note. CMRI: Cardiac Magnetic Resonance Imaging (all T2 sequence reported in this table), **CCTA:** Coronary Computed Tomography Angiography, **LGE:** Late gadolinium enhancement, **LVEF:** Left ventricle ejection fraction, **NR:** Not reported, **GLS:** global longitudinal strain.

Reference

1. Albert E, Aurigemma G, Saucedo J, Gerson DS. Myocarditis following COVID-19 vaccination. Radiol Case Rep. 2021;16(8):2142-5. Epub 2021/05/25. doi: 10.1016/j.radcr.2021.05.033. PubMed PMID: 34025885; PubMed Central PMCID: PMCPMC8130498.
2. Ammirati E, Cavalotti C, Milazzo A, Pedrotti P, Soriano F, Schroeder JW, et al. Temporal Relation Between Second Dose BNT162b2 mRNA Covid-19 Vaccine and Cardiac involvement in a Patient with Previous SARS-COV-2 Infection. Int J Cardiol Heart Vasc. 2021:100778. Epub 2021/04/13. doi: 10.1016/j.ijcha.2021.100778. PubMed PMID: 33842684; PubMed Central PMCID: PMCPMC8020086 for publication. EA received honoraria for participation to advisory board from Kiniksa Pharmaceutical in the last 3 years.
3. D'Angelo T, Cattafi A, Carerj ML, Booz C, Ascenti G, Cicero G, et al. Myocarditis After SARS-CoV-2 Vaccination: A Vaccine-Induced Reaction? Can J Cardiol. 2021;37(10):1665-7. Epub 2021/06/13. doi: 10.1016/j.cjca.2021.05.010. PubMed PMID: 34118375; PubMed Central PMCID: PMCPMC8187737.
4. Deb A, Abdelmalek J, Iwuji K, Nugent K. Acute Myocardial Injury Following COVID-19 Vaccination: A Case Report and Review of Current Evidence from Vaccine Adverse Events Reporting System Database. J Prim Care Community Health. 2021;12:21501327211029230. Epub 2021/07/06. doi: 10.1177/21501327211029230. PubMed PMID: 34219532; PubMed Central PMCID: PMCPMC8255555.
5. Bautista García J, Peña Ortega P, Bonilla Fernández JA, Cárdenes León A, Ramírez Burgos L, Caballero Dorta E. Acute myocarditis after administration of the BNT162b2 vaccine against COVID-19. Rev Esp Cardiol (Engl Ed). 2021;74(9):812-4. Epub 2021/05/18. doi: 10.1016/j.rec.2021.04.005. PubMed PMID: 33994339; PubMed Central PMCID: PMCPMC8075838.
6. Habib MB, Hamamyh T, Elyas A, Altermanini M, Elhassan M. Acute myocarditis following administration of BNT162b2 vaccine. IDCases. 2021;25:e01197. Epub 2021/07/01. doi: 10.1016/j.idcr.2021.e01197. PubMed PMID: 34189042; PubMed Central PMCID: PMCPMC8220234.
7. Shaw KE, Cavalcante JL, Han BK, Gössl M. Possible Association Between COVID-19 Vaccine and Myocarditis: Clinical and CMR Findings. JACC Cardiovasc Imaging. 2021;14(9):1856-61. Epub 2021/07/12. doi: 10.1016/j.jcmg.2021.06.002. PubMed PMID: 34246586; PubMed Central PMCID: PMCPMC8245050.
8. Watkins K, Griffin G, Septaric K, Simon EL. Myocarditis after BNT162b2 vaccination in a healthy male. Am J Emerg Med. 2021;50:815.e1-.e2. Epub 2021/07/08. doi: 10.1016/j.ajem.2021.06.051. PubMed PMID: 34229940; PubMed Central PMCID: PMCPMC8238643.
9. Singh B, Kaur P, Cedeno L, Brahimi T, Patel P, Virk H, et al. COVID-19 mRNA Vaccine and Myocarditis. Eur J Case Rep Intern Med. 2021;8(7):002681. Epub 2021/07/17. doi: 10.12890/2021_002681. PubMed PMID: 34268277; PubMed Central PMCID: PMCPMC8276934.

10. Muthukumar A, Narasimhan M, Li QZ, Mahimainathan L, Hitto I, Fuda F, et al. In-Depth Evaluation of a Case of Presumed Myocarditis After the Second Dose of COVID-19 mRNA Vaccine. Circulation. 2021;144(6):487-98. Epub 2021/06/17. doi: 10.1161/circulationaha.121.056038. PubMed PMID: 34133883; PubMed Central PMCID: PMCPMC8340727.
11. Minocha PK, Better D, Singh RK, Hoque T. Recurrence of Acute Myocarditis Temporally Associated with Receipt of the mRNA Coronavirus Disease 2019 (COVID-19) Vaccine in a Male Adolescent. J Pediatr. 2021;238:321-3. Epub 2021/06/25. doi: 10.1016/j.jpeds.2021.06.035. PubMed PMID: 34166671; PubMed Central PMCID: PMCPMC8216855.
12. McLean K, Johnson TJ. Myopericarditis in a previously healthy adolescent male following COVID-19 vaccination: A case report. Acad Emerg Med. 2021;28(8):918-21. Epub 2021/06/17. doi: 10.1111/acem.14322. PubMed PMID: 34133825; PubMed Central PMCID: PMCPMC8441784.
13. Dickey JB, Albert E, Badr M, Laraja KM, Sena LM, Gerson DS, et al. A Series of Patients With Myocarditis Following SARS-CoV-2 Vaccination With mRNA-1279 and BNT162b2. JACC Cardiovasc Imaging. 2021;14(9):1862-3. Epub 2021/07/12. doi: 10.1016/j.jcmg.2021.06.003. PubMed PMID: 34246585; PubMed Central PMCID: PMCPMC8219373.
14. Kim HW, Jenista ER, Wendell DC, Azevedo CF, Campbell MJ, Darty SN, et al. Patients With Acute Myocarditis Following mRNA COVID-19 Vaccination. JAMA Cardiol. 2021;6(10):1196-201. Epub 2021/06/30. doi: 10.1001/jamacardio.2021.2828. PubMed PMID: 34185046; PubMed Central PMCID: PMCPMC8243258 Longeeveron outside the submitted work. Dr R. J. Kim reported holding equity interest in Heart Imaging Technologies and grants from Siemens outside the submitted work. No other disclosures were reported.
15. Larson KF, Ammirati E, Adler ED, Cooper LT, Jr., Hong KN, Saponara G, et al. Myocarditis After BNT162b2 and mRNA-1273 Vaccination. Circulation. 2021;144(6):506-8. Epub 2021/06/17. doi: 10.1161/circulationaha.121.055913. PubMed PMID: 34133884; PubMed Central PMCID: PMCPMC8340725.
16. Vidula MK, Ambrose M, Glassberg H, Chokshi N, Chen T, Ferrari VA, et al. Myocarditis and Other Cardiovascular Complications of the mRNA-Based COVID-19 Vaccines. Cureus. 2021;13(6):e15576. Epub 2021/07/20. doi: 10.7759/cureus.15576. PubMed PMID: 34277198; PubMed Central PMCID: PMCPMC8270057.
17. Mansour J, Short RG, Bhalla S, Woodard PK, Verma A, Robinson X, et al. Acute myocarditis after a second dose of the mRNA COVID-19 vaccine: a report of two cases. Clin Imaging. 2021;78:247-9. Epub 2021/06/25. doi: 10.1016/j.clinimag.2021.06.019. PubMed PMID: 34166884; PubMed Central PMCID: PMCPMC8216670.
18. Marshall M, Ferguson ID, Lewis P, Jaggi P, Gagliardo C, Collins JS, et al. Symptomatic Acute Myocarditis in 7 Adolescents After Pfizer-BioNTech COVID-19 Vaccination. Pediatrics. 2021;148(3). Epub 2021/06/06. doi: 10.1542/peds.2021-052478. PubMed PMID: 34088762.
19. Montgomery J, Ryan M, Engler R, Hoffman D, McClenathan B, Collins L, et al. Myocarditis Following Immunization With mRNA COVID-19 Vaccines in Members of the US Military. JAMA Cardiol. 2021;6(10):1202-6. Epub 2021/06/30. doi: 10.1001/jamacardio.2021.2833. PubMed PMID: 34185045; PubMed Central PMCID: PMCPMC8243257 Myers Squibb, Cantargia, CardioPath, Kiniksa Pharmaceuticals, and Cardiol Therapeutics. No other disclosures were reported.

20. Abu Mouch S, Roguin A, Hellou E, Ishai A, Shoshan U, Mahamid L, et al. Myocarditis following COVID-19 mRNA vaccination. Vaccine. 2021;39(29):3790-3. Epub 2021/06/08. doi: 10.1016/j.vaccine.2021.05.087. PubMed PMID: 34092429; PubMed Central PMCID: PMCPMC8162819.
21. Nevet A. Acute myocarditis associated with anti-COVID-19 vaccination. Clin Exp Vaccine Res. 2021;10(2):196-7. Epub 2021/07/06. doi: 10.7774/cevr.2021.10.2.196. PubMed PMID: 34222133; PubMed Central PMCID: PMCPMC8217579.
22. Rosner CM, Genovese L, Tehrani BN, Atkins M, Bakhshi H, Chaudhri S, et al. Myocarditis Temporally Associated With COVID-19 Vaccination. Circulation. 2021;144(6):502-5. Epub 2021/06/17. doi: 10.1161/circulationaha.121.055891. PubMed PMID: 34133885; PubMed Central PMCID: PMCPMC8340723.
23. Schauer J, Buddhe S, Colyer J, Sagiv E, Law Y, Mallenahalli Chikkabyrappa S, et al. Myopericarditis After the Pfizer Messenger Ribonucleic Acid Coronavirus Disease Vaccine in Adolescents. J Pediatr. 2021;238:317-20. Epub 2021/07/07. doi: 10.1016/j.jpeds.2021.06.083. PubMed PMID: 34228985; PubMed Central PMCID: PMCPMC8253718.
24. Park J, Brekke DR, Bratincsak A. Self-limited myocarditis presenting with chest pain and ST segment elevation in adolescents after vaccination with the BNT162b2 mRNA vaccine. Cardiol Young. 2022;32(1):146-9. Epub 2021/06/29. doi: 10.1017/s1047951121002547. PubMed PMID: 34180390.
25. Cereda A, Conca C, Barbieri L, Ferrante G, Tumminello G, Lucreziotti S, et al. Acute myocarditis after the second dose of SARS-CoV-2 vaccine: Serendipity or atypical causal relationship? Anatol J Cardiol. 2021;25(7):522-3. Epub 2021/07/09. doi: 10.5152/AnatolJCardiol.2021.99. PubMed PMID: 34236331; PubMed Central PMCID: PMCPMC8274899.
26. Williams CB, Choi J-i, Hosseini F, Roberts J, Ramanathan K, Ong K. Acute Myocarditis Following mRNA-1273 SARS-CoV-2 Vaccination. CJC Open. 2021;3(11):1410-2. doi: 10.1016/j.cjco.2021.07.008.
27. Starekova J, Bluemke DA, Bradham WS, Grist TM, Schiebler ML, Reeder SB. Myocarditis Associated with mRNA COVID-19 Vaccination. Radiology. 2021;301(2):E409-e11. Epub 2021/07/21. doi: 10.1148/radiol.2021211430. PubMed PMID: 34282971; PubMed Central PMCID: PMCPMC8574056 D.A.B. is Editor of Radiology. W.S.B. disclosed no relevant relationships. T.M.G. is member of Bracco Advisory Board; disclosed personal ownership interest/stockholder in Elucent, Histosonics, and Shine Medical; disclosed institutional research support from GE Healthcare, Bracco Diagnostics, Siemens, Hologic, and Change Healthcare. M.L.S. has patents (planned, issued, or pending) on CT of mucus plug coring; disclosed leadership or fiduciary role with Fleischner Society; is shareholder in Healthmyne, X-Vax, and Stemina Biomarker Discovery; is member of the Radiology editorial board. S.B.R. disclosed ownership interests in Calimetrix, Reveal Pharmaceuticals, Cellectar Biosciences, Elucent Medical, and HeartVista; the University of Wisconsin receives research support from GE Healthcare and Bracco Diagnostics.
28. Nassar M, Nso N, Gonzalez C, Lakhdar S, Alshamam M, Elshafey M, et al. COVID-19 vaccine-induced myocarditis: Case report with literature review. Diabetes Metab Syndr. 2021;15(5):102205. Epub 2021/07/23. doi: 10.1016/j.dsx.2021.102205. PubMed PMID: 34293552; PubMed Central PMCID: PMCPMC8270733.
29. Di Tano G ML, Calvaruso EV, Danzi GB. Recurrent Myocarditis after the First Dose of SARS-CoV-2 mRNA- 1273 Vaccine. Ann Clin Case Rep. 2021(6):2018.

30. Khogali F, Abdelrahman R. Unusual Presentation of Acute Perimyocarditis Following SARS-COV-2 mRNA-1237 Moderna Vaccination. Cureus. 2021;13(7):e16590. Epub 2021/08/28. doi: 10.7759/cureus.16590. PubMed PMID: 34447639; PubMed Central PMCID: PMCPMC8381757.
31. Tano E, San Martin S, Girgis S, Martinez-Fernandez Y, Sanchez Vegas C. Perimyocarditis in Adolescents After Pfizer-BioNTech COVID-19 Vaccine. J Pediatric Infect Dis Soc. 2021;10(10):962-6. Epub 2021/07/29. doi: 10.1093/jpids/piab060. PubMed PMID: 34319393; PubMed Central PMCID: PMCPMC8344528.
32. Isaak A, Feisst A, Luetkens JA. Myocarditis Following COVID-19 Vaccination. Radiology. 2021;301(1):E378-e9. Epub 2021/08/04. doi: 10.1148/radiol.2021211766. PubMed PMID: 34342500; PubMed Central PMCID: PMCPMC8369878.
33. Verma AK, Lavine KJ, Lin CY. Myocarditis after Covid-19 mRNA Vaccination. N Engl J Med. 2021;385(14):1332-4. Epub 2021/08/19. doi: 10.1056/NEJMc2109975. PubMed PMID: 34407340; PubMed Central PMCID: PMCPMC8385564.
34. Diaz GA, Parsons GT, Gering SK, Meier AR, Hutchinson IV, Robicsek A. Myocarditis and Pericarditis After Vaccination for COVID-19. Jama. 2021;326(12):1210-2. Epub 2021/08/05. doi: 10.1001/jama.2021.13443. PubMed PMID: 34347001; PubMed Central PMCID: PMCPMC8340007 research support from Gilead Sciences, Regeneron, Roche, Boehringer Ingelheim, and Edesa Biotech and scientific advisory board membership for Safeology. No other disclosures were reported.
35. Levin D, Shimon G, Fadlon-Derai M, Gershovitz L, Shovali A, Sebbag A, et al. Myocarditis following COVID-19 vaccination - A case series. Vaccine. 2021;39(42):6195-200. Epub 2021/09/19. doi: 10.1016/j.vaccine.2021.09.004. PubMed PMID: 34535317; PubMed Central PMCID: PMCPMC8416687.
36. Shumkova M, Vassilev D, Karamfiloff K, Ivanova R, Stoyanova K, Yaneva-Sirakova T, et al. Acute myocarditis associated with the Pfizer/BioNTech vaccine. Kardiol Pol. 2021;79(11):1282-3. Epub 2021/08/24. doi: 10.33963/KP.a2021.0095. PubMed PMID: 34424994.
37. Cimaglia P, Tolomeo P, Rapezzi C. Acute myocarditis after SARS-CoV-2 vaccination in a 24-year-old man. Rev Port Cardiol. 2022;41(1):71-2. Epub 2021/08/03. doi: 10.1016/j.repc.2021.07.005. PubMed PMID: 34334935; PubMed Central PMCID: PMCPMC8302854.
38. Dionne A, Sperotto F, Chamberlain S, Baker AL, Powell AJ, Prakash A, et al. Association of Myocarditis With BNT162b2 Messenger RNA COVID-19 Vaccine in a Case Series of Children. JAMA Cardiol. 2021;6(12):1446-50. Epub 2021/08/11. doi: 10.1001/jamacardio.2021.3471. PubMed PMID: 34374740; PubMed Central PMCID: PMCPMC8356143.
39. Ehrlich P, Klingel K, Ohlmann-Knafo S, Hüttinger S, Sood N, Pickuth D, et al. Biopsy-proven lymphocytic myocarditis following first mRNA COVID-19 vaccination in a 40-year-old male: case report. Clin Res Cardiol. 2021;110(11):1855-9. Epub 2021/09/06. doi: 10.1007/s00392-021-01936-6. PubMed PMID: 34487236.
40. Gautam N, Saluja P, Fudim M, Jambhekar K, Pandey T, Al'Aref S. A Late Presentation of COVID-19 Vaccine-Induced Myocarditis. Cureus. 2021;13(9):e17890. Epub 2021/10/19. doi: 10.7759/cureus.17890. PubMed PMID: 34660088; PubMed Central PMCID: PMCPMC8504680.

41. Kim IC, Kim H, Lee HJ, Kim JY, Kim JY. Cardiac Imaging of Acute Myocarditis Following COVID-19 mRNA Vaccination. J Korean Med Sci. 2021;36(32):e229-e. doi: 10.3346/jkms.2021.36.e229. PubMed PMID: 34402228.
42. Jain SS, Steele JM, Fonseca B, Huang S, Shah S, Maskatia SA, et al. COVID-19 Vaccination-Associated Myocarditis in Adolescents. Pediatrics. 2021;148(5). Epub 2021/08/15. doi: 10.1542/peds.2021-053427. PubMed PMID: 34389692.
43. King WW, Petersen MR, Matar RM, Budweg JB, Cuervo Pardo L, Petersen JW. Myocarditis following mRNA vaccination against SARS-CoV-2, a case series. Am Heart J Plus. 2021;8:100042. Epub 2021/08/17. doi: 10.1016/j.ahjo.2021.100042. PubMed PMID: 34396358; PubMed Central PMCID: PMCPMC8349733.
44. Matta A, Kallamadi R, Matta D, Bande D. Post-mRNA COVID-19 Vaccination Myocarditis. Eur J Case Rep Intern Med. 2021;8(8):002769. Epub 2021/09/17. doi: 10.12890/2021_002769. PubMed PMID: 34527626; PubMed Central PMCID: PMCPMC8436846.
45. Patel YR, Louis DW, Atalay M, Agarwal S, Shah NR. Cardiovascular magnetic resonance findings in young adult patients with acute myocarditis following mRNA COVID-19 vaccination: a case series. Journal of Cardiovascular Magnetic Resonance. 2021;23(1):101. doi: 10.1186/s12968-021-00795-4.
46. Patrignani A, Schicchi N, Calcagnoli F, Falchetti E, Ciampani N, Argalia G, et al. Acute myocarditis following Comirnaty vaccination in a healthy man with previous SARS-CoV-2 infection. Radiol Case Rep. 2021;16(11):3321-5. Epub 2021/08/10. doi: 10.1016/j.radcr.2021.07.082. PubMed PMID: 34367386; PubMed Central PMCID: PMCPMC8326008.
47. Sulemankhil I, Abdelrahman M, Negi SI. Temporal association between the COVID-19 Ad26.COV2.S vaccine and acute myocarditis: A case report and literature review. Cardiovasc Revasc Med. 2021. Epub 2021/08/24. doi: 10.1016/j.carrev.2021.08.012. PubMed PMID: 34420869; PubMed Central PMCID: PMCPMC8364889.
48. Tailor PD, Feighery AM, El-Sabawi B, Prasad A. Case report: acute myocarditis following the second dose of mRNA-1273 SARS-CoV-2 vaccine. Eur Heart J Case Rep. 2021;5(8):ytab319. Epub 2021/09/14. doi: 10.1093/ehjcr/ytab319. PubMed PMID: 34514306; PubMed Central PMCID: PMCPMC8422333.
49. Ujueta F, Azimi R, Lozier MR, Poppiti R, Ciment A. Lymphohistocytic myocarditis after Ad26.COV2.S viral vector COVID-19 vaccination. Int J Cardiol Heart Vasc. 2021;36:100869. Epub 2021/09/14. doi: 10.1016/j.ijcha.2021.100869. PubMed PMID: 34514078; PubMed Central PMCID: PMCPMC8421108 personal relationships that could have appeared to influence the work reported in this paper.
50. Hung YP, Sun KS. A case of myopericarditis with pleuritis following AstraZeneca Covid-19 vaccination. Qjm. 2022;114(12):879-81. Epub 2021/11/07. doi: 10.1093/qjmed/hcab278. PubMed PMID: 34741522; PubMed Central PMCID: PMCPMC8689895.
51. Kaneta K, Yokoi K, Jojima K, Kotooka N, Node K. Young Male With Myocarditis Following mRNA-1273 Vaccination Against Coronavirus Disease-2019 (COVID-19). Circ J. 2022;86(4):721. Epub 2021/11/09. doi: 10.1253/circj.CJ-21-0818. PubMed PMID: 34744118.

52. Kaul R, Sreenivasan J, Goel A, Malik A, Bandyopadhyay D, Jin C, et al. Myocarditis following COVID-19 vaccination. Int J Cardiol Heart Vasc. 2021;36:100872. Epub 2021/09/28. doi: 10.1016/j.ijcha.2021.100872. PubMed PMID: 34568540; PubMed Central PMCID: PMCPMC8450283 personal relationships that could have appeared to influence the work reported in this paper.
53. Kim D, Choi JH, Jang JY, So O, Cho E, Choi H, et al. A Case Report for Myopericarditis after BNT162b2 COVID-19 mRNA Vaccination in a Korean Young Male. J Korean Med Sci. 2021;36(39):e277. Epub 2021/10/13. doi: 10.3346/jkms.2021.36.e277. PubMed PMID: 34636504; PubMed Central PMCID: PMCPMC8506415.
54. Koizumi T, Awaya T, Yoshioka K, Kitano S, Hayama H, Amemiya K, et al. Myocarditis after COVID-19 mRNA vaccines. Qjm. 2021;114(10):741-3. Epub 2021/09/22. doi: 10.1093/qjmed/hcab244. PubMed PMID: 34546329.
55. Maki H, Aikawa T, Ibe T, Oyama-Manabe N, Fujita H. Biventricular systolic dysfunction in acute myocarditis after SARS-CoV-2 mRNA-1273 vaccination. Eur Heart J Cardiovasc Imaging. 2022;23(2):e87. Epub 2021/10/04. doi: 10.1093/ehjci/jeab206. PubMed PMID: 34601566.
56. Miqdad MA, Nasser H, Alshehri A, Mourad AR. Acute Myocarditis Following the Administration of the Second BNT162b2 COVID-19 Vaccine Dose. Cureus. 2021;13(10):e18880. Epub 2021/11/23. doi: 10.7759/cureus.18880. PubMed PMID: 34804729; PubMed Central PMCID: PMCPMC8599115.
57. Nguyen TD, Mall G, Westphal JG, Weingärtner O, Möbius-Winkler S, Schulze PC. Acute myocarditis after COVID-19 vaccination with mRNA-1273 in a patient with former SARS-CoV-2 infection. ESC Heart Fail. 2021;8(6):4710-4. Epub 2021/09/19. doi: 10.1002/ehf2.13613. PubMed PMID: 34536056; PubMed Central PMCID: PMCPMC8652969.
58. Onderko L, Starobin B, Riviere AE, Hohl PK, Phillips CT, Morgan RB, et al. Myocarditis in the Setting of Recent COVID-19 Vaccination. Case Rep Cardiol. 2021;2021:6806500. Epub 2021/10/30. doi: 10.1155/2021/6806500. PubMed PMID: 34712497; PubMed Central PMCID: PMCPMC8548171.
59. Pareek M, Asnes. Myopericarditis after Covid-19 Vaccination – a Case Series. 2021. doi: Myopericarditis after Covid-19 Vaccination – a Case Series.
60. Perez Y, Levy ER, Joshi AY, Virk A, Rodriguez-Porcel M, Johnson M, et al. Myocarditis Following COVID-19 mRNA Vaccine: A Case Series and Incidence Rate Determination. Clin Infect Dis. 2021. Epub 2021/11/05. doi: 10.1093/cid/ciab926. PubMed PMID: 34734240; PubMed Central PMCID: PMCPMC8767838.
61. Sakaguchi S, Fujimoto N, Ichikawa K, Izumi D, Katsuta K, Takafuji M, et al. Myopericarditis After COVID-19 mRNA Vaccination. Circ J. 2022;86(3):472. Epub 2021/10/01. doi: 10.1253/circj.CJ-21-0683. PubMed PMID: 34588400.
62. Schmitt P, Demoulin R, Poyet R, Capilla E, Rohel G, Pons F, et al. Acute Myocarditis after COVID-19 vaccination: A case report. Rev Med Interne. 2021;42(11):797-800. Epub 2021/11/07. doi: 10.1016/j.revmed.2021.10.003. PubMed PMID: 34740463; PubMed Central PMCID: PMCPMC8523482.
63. Sivakumaran P, Sunny J, Tsagkridi A, Khanji MY. Myopericarditis following SARS-CoV-2 mRNA vaccine: the role of cardiac biomarkers and multimodality imaging. Eur Heart J Cardiovasc Imaging. 2022;23(3):e134. Epub 2021/09/07. doi: 10.1093/ehjci/jeab183. PubMed PMID: 34487161; PubMed Central PMCID: PMCPMC8499913.

64. Viskin D, Topilsky Y, Aviram G, Mann T, Sadon S, Hadad Y, et al. Myocarditis Associated With COVID-19 Vaccination: Echocardiography, Cardiac Tomography, and Magnetic Resonance Imaging Findings. Circ Cardiovasc Imaging. 2021;14(9):e013236. Epub 2021/08/26. doi: 10.1161/circimaging.121.013236. PubMed PMID: 34428917; PubMed Central PMCID: PMCPMC8478100.
65. Vollmann D, Eiffert H, Schuster A. Acute Perimyocarditis Following First Dose of mRNA Vaccine Against COVID-19. Dtsch Arztebl Int. 2021;118(31-32):546. Epub 2021/09/14. doi: 10.3238/arztebl.m2021.0288. PubMed PMID: 34515024; PubMed Central PMCID: PMCPMC8422910.
66. Wilson H, Norris MD, Frosch O, Agarwal PP. Late Gadolinium Enhancement after COVID-19 Vaccination. Radiol Cardiothorac Imaging. 2021;3(5):e210199-e. doi: 10.1148/ryct.2021210199. PubMed PMID: 34778788.
67. Shiyovich A, Witberg G, Aviv Y, Eisen A, Orvin K, Wiessman M, et al. Myocarditis following COVID-19 vaccination: magnetic resonance imaging study. Eur Heart J Cardiovasc Imaging. 2021. Epub 2021/11/06. doi: 10.1093/ehjci/jeab230. PubMed PMID: 34739045.
68. Hasnie AA, Hasnie UA, Patel N, Aziz MU, Xie M, Lloyd SG, et al. Perimyocarditis following first dose of the mRNA-1273 SARS-CoV-2 (Moderna) vaccine in a healthy young male: a case report. BMC Cardiovascular Disorders. 2021;21(1):375. doi: 10.1186/s12872-021-02183-3.
69. Habedank D, Lagast A, Novoa-Usme M, Atmowihardjo I. A case of myocarditis in a 60-year-old man 48 h after mRNA vaccination against SARS-CoV2. Clin Res Cardiol. 2022;111(2):230-2. Epub 2021/11/04. doi: 10.1007/s00392-021-01946-4. PubMed PMID: 34731321; PubMed Central PMCID: PMCPMC8564591.
70. Angel Goenawan FK, Kenneth Schwartz and Seema D'Souza. Recurrent mRNA (BNT162b2) Covid-19 Vaccine-Associated Pericarditis in an Elderly Man with Multiple Comorbidities. American Journal of Medical Case Reports. 2021;9(12):709-13. doi: doi: 10.12691/AJMCR-9-12-12.
71. Fleming-Nouri A, Haimovich AD, Yang D, Schulz WL, Coppi A, Taylor RA. Myopericarditis in young adults presenting to the emergency department after receiving a second COVID-19 mRNA vaccine. Acad Emerg Med. 2021;28(7):802-5. Epub 2021/07/27. doi: 10.1111/acem.14307. PubMed PMID: 34310793; PubMed Central PMCID: PMCPMC8441914.
72. Das BB, Kohli U, Ramachandran P, Nguyen HH, Greil G, Hussain T, et al. Myopericarditis after messenger RNA Coronavirus Disease 2019 Vaccination in Adolescents 12 to 18 Years of Age. J Pediatr. 2021;238:26-32.e1. Epub 2021/08/03. doi: 10.1016/j.jpeds.2021.07.044. PubMed PMID: 34339728; PubMed Central PMCID: PMCPMC8321962.
73. Chen DH, Arefin AR, Joshi A, Khanji MY. Myopericarditis in a teenager following first mRNA COVID vaccine dose: the role of multi-parametric cardiovascular magnetic resonance. Eur Heart J Case Rep. 2021;5(10):ytab371. Epub 2021/10/09. doi: 10.1093/ehjcr/ytab371. PubMed PMID: 34622131; PubMed Central PMCID: PMCPMC8493007.
74. Chelala L, Jeudy J, Hossain R, Rosenthal G, Pietris N, White CS. Cardiac MRI Findings of Myocarditis After COVID-19 mRNA Vaccination in Adolescents. AJR Am J Roentgenol. 2022;218(4):651-7. Epub 2021/10/28. doi: 10.2214/ajr.21.26853. PubMed PMID: 34704459.

75. Boursier C, Chevalier E, Filippetti L, Imbert L, Roch V, Huttin O, et al. (68)Ga-DOTATOC digital-PET imaging of inflammatory cell infiltrates in myocarditis following COVID-19 vaccination. Eur J Nucl Med Mol Imaging. 2022;49(4):1433-4. Epub 2021/11/09. doi: 10.1007/s00259-021-05609-4. PubMed PMID: 34746968; PubMed Central PMCID: PMCPMC8572651.
76. Badshah M, Shriver J, Rynders B, Sjovold A, Shaukat MHS, Rajpurohit N. MODERNA mRNA-1273 vaccine-associated myopericarditis in a patient with a subclinical autoimmune predisposition. J Cardiol Cases. 2021;24(5):227-9. Epub 2021/12/07. doi: 10.1016/j.jccase.2021.09.007. PubMed PMID: 34868402; PubMed Central PMCID: PMCPMC8617476.
77. Azir M, Inman B, Webb J, Tannenbaum L. STEMI Mimic: Focal Myocarditis in an Adolescent Patient After mRNA COVID-19 Vaccine. J Emerg Med. 2021;61(6):e129-e32. Epub 2021/11/11. doi: 10.1016/j.jemermed.2021.09.017. PubMed PMID: 34756746; PubMed Central PMCID: PMCPMC8469220.
78. Ambati S, Colon M, Mihic M, Sanchez J, Bakar A. Acute Myopericarditis after COVID-19 Vaccine in Teenagers. Case Rep Cardiol. 2021;2021:8268755. Epub 2021/10/01. doi: 10.1155/2021/8268755. PubMed PMID: 34589238; PubMed Central PMCID: PMCPMC8476255.
79. Sokolska JM, Kurcz J, Kosmala W. Every rose has its thorns - acute myocarditis following COVID-19 vaccination. Kardiol Pol. 2021;79(10):1153-4. Epub 2021/08/01. doi: 10.33963/KP.a2021.0075. PubMed PMID: 34331307.
80. Lazaros G, Anastassopoulou C, Hatziantoniou S, Kalos T, Soulaidopoulos S, Lazarou E, et al. A case series of acute pericarditis following COVID-19 vaccination in the context of recent reports from Europe and the United States. Vaccine. 2021;39(45):6585-90. Epub 2021/10/13. doi: 10.1016/j.vaccine.2021.09.078. PubMed PMID: 34635376; PubMed Central PMCID: PMCPMC8491922.
81. Facetti S. GM, Vecchi AL. Acute myocarditis in a young adult two days after Pfizer vaccine. G Ital Cardiol. 2021;22(11):891-3. doi: doi 10.1714 / 3689.36746.
82. Meyer-Szary J, Bazgier M, Lubocka P, Dorniak K, Sabiniewicz R. Cardiac magnetic resonance characteristics of acute myocarditis occurring after mRNA-based COVID-19 vaccines immunization. Cardiol J. 2022;29(1):160-2. Epub 2021/11/18. doi: 10.5603/CJ.a2021.0152. PubMed PMID: 34787887; PubMed Central PMCID: PMCPMC8890411.
83. Aikawa T, Ogino J, Kita Y, Funayama N. Myocardial microthrombi after COVID-19 mRNA vaccination. Eur Heart J. 2021;42(43):4501. Epub 2021/10/09. doi: 10.1093/eurheartj/ehab727. PubMed PMID: 34624077; PubMed Central PMCID: PMCPMC8599010.
84. Bricoli S, Cacciola G, Barocelli F, Guerra C, Zardini M. 553 Myocarditis after COVID-19 vaccination—a case series. European Heart Journal Supplements. 2021;23(Supplement_G). doi: 10.1093/eurheartj/suab135.008.
85. Chachar TS, Yousuf N, Sulaibikh L, Abdulqader F, Alqahtani M. First Report of Acute Myocarditis Post-Pfizer-BioNTech COVID-19 Vaccination in the Kingdom of Bahrain. Cureus. 2021;13(12):e20313. Epub 2022/01/15. doi: 10.7759/cureus.20313. PubMed PMID: 35028213; PubMed Central PMCID: PMCPMC8747994.

86. Eggebrecht H, Breitbart P, Koch A, Nowak B, Walther C, Voigtländer T, et al. Trends in ambulatory cardiology consultations for suspected myocarditis after COVID-19 vaccination. Clin Res Cardiol. 2022;111(2):237-9. Epub 2021/11/24. doi: 10.1007/s00392-021-01974-0. PubMed PMID: 34812930; PubMed Central PMCID: PMCPMC8608850 of interest.
87. Istampoulouoglou I, Dimitriou G, Späni S, Christ A, Zimmermanns B, Koechlin S, et al. Myocarditis and pericarditis in association with COVID-19 mRNA-vaccination: cases from a regional pharmacovigilance centre. Glob Cardiol Sci Pract. 2021;2021(3):e202118. Epub 2021/11/23. doi: 10.21542/gcsp.2021.18. PubMed PMID: 34805376; PubMed Central PMCID: PMCPMC8587334.
88. Lim Y, Kim MC, Kim KH, Jeong I-S, Cho YS, Choi YD, et al. Case Report: Acute Fulminant Myocarditis and Cardiogenic Shock After Messenger RNA Coronavirus Disease 2019 Vaccination Requiring Extracorporeal Cardiopulmonary Resuscitation. Frontiers in Cardiovascular Medicine. 2021;8. doi: 10.3389/fcvm.2021.758996.
89. McCullough J, McCullough JP, Korlipara G, Kaell A. Myocarditis Post Moderna Vaccination: Review of Criteria for Diagnosis. Cureus. 2021;13(11):e19633. Epub 2021/12/28. doi: 10.7759/cureus.19633. PubMed PMID: 34956759; PubMed Central PMCID: PMCPMC8675599.
90. Murakami Y, Shinohara M, Oka Y, Wada R, Noike R, Ohara H, et al. Myocarditis Following a COVID-19 Messenger RNA Vaccination: A Japanese Case Series. Intern Med. 2022;61(4):501-5. Epub 2021/11/30. doi: 10.2169/internalmedicine.8731-21. PubMed PMID: 34840235; PubMed Central PMCID: PMCPMC8907764.
91. Nagasaka T, Koitabashi N, Ishibashi Y, Aihara K, Takama N, Ohyama Y, et al. Acute Myocarditis Associated with COVID-19 Vaccination: A Case Report. J Cardiol Cases. 2021. Epub 2021/12/09. doi: 10.1016/j.jccase.2021.11.006. PubMed PMID: 34876937; PubMed Central PMCID: PMCPMC8639400.
92. Parmar K, Mekraksakit P, Del Rio-Pertuz G, Sethi P, Motes A, Hughes M, et al. Myocarditis following COVID-19 mRNA vaccination. Baylor University Medical Center Proceedings. 2022;35(2):209-13. doi: 10.1080/08998280.2021.1990743.
93. Singh R, Chakrabarti SS, Gambhir IS, Verma A, Kumar I, Ghosh S, et al. Acute Cardiac Events After ChAdOx1 nCoV-19 Corona Virus Vaccine: Report of Three Cases. Am J Ther. 2022. Epub 2022/02/18. doi: 10.1097/mjt.0000000000001472. PubMed PMID: 35175717.
94. Takeda M, Ishio N, Shoji T, Mori N, Matsumoto M, Shikama N. Eosinophilic Myocarditis Following Coronavirus Disease 2019 (COVID-19) Vaccination. Circulation Journal. 2021;advpub. doi: 10.1253/circj.CJ-21-0935.
95. Tinoco M, Leite S, Faria B, Cardoso S, Von Hafe P, Dias G, et al. Perimyocarditis Following COVID-19 Vaccination. Clin Med Insights Cardiol. 2021;15:11795468211056634. Epub 2021/12/07. doi: 10.1177/11795468211056634. PubMed PMID: 34866957; PubMed Central PMCID: PMCPMC8637777.
96. Ashutosh T, Sankha Shubhra C, Prasan Kumar P, Gaurav K, Upinder K. Research Square. 2022. doi: 10.21203/rs.3.rs-806335/v1.

97. Di Dedda EA, Barison A, Aquaro GD, Ismail TF, Hua A, Mantini C, et al. Cardiac magnetic resonance imaging of myocarditis and pericarditis following COVID-19 vaccination: a multicenter collection of 27 cases. Eur Radiol. 2022:1-9. Epub 2022/03/02. doi: 10.1007/s00330-022-08566-0. PubMed PMID: 35230520; PubMed Central PMCID: PMCPMC8886710.
98. Fronza M, Thavendiranathan P, Chan V, Karur GR, Udell JA, Wald RM, et al. Myocardial Injury Pattern at MRI in COVID-19 Vaccine-associated Myocarditis. Radiology. 2022:212559. Epub 2022/02/16. doi: 10.1148/radiol.212559. PubMed PMID: 35166587; PubMed Central PMCID: PMCPMC8856022 Amicus and Medscape. Dr. Thavendiranathan has received speaker's honorarium from Amgen, BI, and Takeda. Dr. Udell has served as a consultant or speaker for AstraZeneca, Bayer, Boehringer Ingelheim-Lilly, Janssen, Merck, Novartis, and Sanofi and has received research grants from AstraZeneca, Amgen, Bayer, Boehringer Ingelheim-Lilly and Janssen.
99. Bews H, Bryson A, Bortoluzzi T, Tam JW, Jassal DS. COVID-19 vaccination induced myopericarditis: an imager's perspective. CJC Open. 2022. Epub 2022/02/08. doi: 10.1016/j.cjco.2022.01.007. PubMed PMID: 35128370; PubMed Central PMCID: PMCPMC8800170.
100. Manfredi R, Bianco F, Bucciarelli V, Ciliberti G, Guerra F, Schicchi N, et al. Clinical Profiles and CMR Findings of Young Adults and Pediatrics with Acute Myocarditis Following mRNA COVID-19 Vaccination: A Case Series. Vaccines. 2022;10(2):169. PubMed PMID: doi:10.3390/vaccines10020169.
101. Sharff KA, Dancoes DM, Longueil JL, Lewis PF, Johnson ES. Myopericarditis After COVID-19 Booster Dose Vaccination. American Journal of Cardiology. doi: 10.1016/j.amjcard.2022.02.039.
102. Nunn S, Kersten J, Tadic M, Wolf A, Gonska B, Hüll E, et al. Case Report: Myocarditis After COVID-19 Vaccination – Case Series and Literature Review. Frontiers in Medicine. 2022;9. doi: 10.3389/fmed.2022.836620.
103. Bengel CP, Kacapor R. A report of two cases of myocarditis following mRNA coronavirus disease 2019 vaccination. Eur Heart J Case Rep. 2022;6(1):ytac004. Epub 2022/02/17. doi: 10.1093/ehjcr/ytac004. PubMed PMID: 35169677; PubMed Central PMCID: PMCPMC8755378.
104. Ohnishi M, Tanaka Y, Nishida S, Sugimoto T. Case report of acute myocarditis after administration of coronavirus disease 2019 vaccine in Japan. Eur Heart J Case Rep. 2022;6(1):ytab534. Epub 2022/01/26. doi: 10.1093/ehjcr/ytab534. PubMed PMID: 35075442; PubMed Central PMCID: PMCPMC8755377.
105. Owuor H, Gikonyo A, Ponoth P, Gikonyo D. Covid Vaccine Induced Myocarditis: A Bolt from the Blue: A Case Report. 2022;7:51-5. doi: 10.21088/jcms.2454.7123.72-321.7.
106. Sano M, Murai R, Kim K, Furukawa Y. Cardiac magnetic resonance findings in acute myocarditis after mRNA COVID-19 vaccination. Journal of cardiology cases. 2022:10.1016/j.jccase.2022.01.015. doi: 10.1016/j.jccase.2022.01.015. PubMed PMID: 35169401.
107. Wong J, Sharma S, Yao JV, Aggarwal A, Grigg L. COVID-19 mRNA vaccine (Comirnaty)-induced myocarditis. Med J Aust. 2022;216(3):122-3. Epub 2022/01/26. doi: 10.5694/mja2.51394. PubMed PMID: 35075640.

108. Wu B, Mittal N, Adler ED, Hong KN. Acute myocarditis after receiving first dose of BNT162b2 mRNA vaccine. Journal of cardiology cases. 2022:10.1016/j.jccase.2021.12.009. doi: 10.1016/j.jccase.2021.12.009. PubMed PMID: 35018202.
109. Yen KC, Ho CT, Chin SC, Su HC, Lee KT, Chu PH. Self-Limited Myocarditis after the First Dose of Coronavirus Disease 2019 Messenger RNA-1273 Vaccine in a Healthy Male. Acta Cardiol Sin. 2022;38(2):210-3. Epub 2022/03/12. doi: 10.6515/acs.202203_38(2).20210912a. PubMed PMID: 35273443; PubMed Central PMCID: PMCPMC8888318.
110. Mohammadi A, Rezaiye M, Goharrizi MASB. Acute Myocarditis Following the Third Dose of SARS-CoV-2 Vaccine; A Case Report. Research Square; 2022.
111. Lee CH, Kong EJ. FDG PET/MRI of Acute Myocarditis After mRNA COVID-19 Vaccination. Clin Nucl Med. 2022. Epub 2022/03/03. doi: 10.1097/rlu.0000000000004123. PubMed PMID: 35234199.
112. Kyaw H, Shajahan S, Gulati A, Synn S, Khurana S, Nazar N, et al. COVID-19 mRNA Vaccine-Associated Myocarditis. Cureus. 2022;14(1):e21009. Epub 2022/02/15. doi: 10.7759/cureus.21009. PubMed PMID: 35154981; PubMed Central PMCID: PMCPMC8820479.
113. Kounis NG, Koniari I, Mplani V, Plotas P, Velissaris D. Hypersensitivity myocarditis and the pathogenetic conundrum of COVID 19 Vaccine Related Myocarditis. Cardiology. 2022. doi: 10.1159/000524224.
114. Van Kerkhove O, Renders F, Leys M. A case of myocarditis following ChAdOx1 nCov-19 vaccination. Acta Cardiol. 2022:1-3. Epub 2022/02/23. doi: 10.1080/00015385.2022.2040825. PubMed PMID: 35189775.
115. Kawakami T, Yahagi K, Sekiguchi M, Ishizawa T, Nonaka H, Setoguchi N, et al. Acute Myocarditis in a Patient Following mRNA-1273 SARS-CoV-2 Vaccination. Intern Med. 2022. Epub 2022/03/08. doi: 10.2169/internalmedicine.9000-21. PubMed PMID: 35249920.
116. Gill J, Mallari AJP, Zahra F. Transient Myopericarditis Following Vaccination for COVID-19. J Med Cases. 2022;13(2):80-4. Epub 2022/03/24. doi: 10.14740/jmc3876. PubMed PMID: 35317093; PubMed Central PMCID: PMCPMC8913003.
117. Agdamag ACC, Gonzalez D, Carlson K, Konety S, McDonald WC, Martin CM, et al. Fulminant myocarditis following coronavirus disease 2019 vaccination: a case report. European Heart Journal - Case Reports. 2022;6(1). doi: 10.1093/ehjcr/ytac007.
118. Fosch X, Serra J, Torres PL, Preda L, González R, Mojer F. Acute myocarditis after a third dose of the BNT162b2 COVID-19 vaccine. Revista espanola de cardiologia (English ed). 2022. doi: 10.1016/j.rec.2022.01.009. PubMed PMID: 35277352.
